# Physics-Constrained Conformal Screening: A Certified Machine Learning Framework for the Rational Design of Dual Glycoepitope-Imprinted Polymers Recognizing CA 19-9 and MUC1-Tn

**DOI:** 10.3390/polym18212699

**Published:** 2026-10-31

**Authors:** Yetkin Senemoğlu, Aytaç Altan

**Affiliations:** 1 Department of Chemistry and Chemical Processing Technologies, Çaycuma Food and Agriculture Vocational School, Zonguldak Bülent Ecevit University, 67900 Zonguldak, Türkiye; yetkin.senemoglu@beun.edu.tr; 2 Department of Electrical and Electronics Engineering, Faculty of Engineering, Zonguldak Bülent Ecevit University, 67100 Zonguldak, Türkiye

**Keywords:** molecularly imprinted polymers, conformal prediction, trustworthy machine learning, distribution-free uncertainty quantification, rational monomer selection, GFN2-xTB, epitope imprinting, CA 19-9, MUC1-Tn, certified in silico screening

## Abstract

The selectivity of a molecularly imprinted polymer (MIP) is decided the moment its functional monomer is chosen, yet that choice is still made largely by trial and error and without any statement of confidence, so a confident but wrong pick can commit months of synthesis before it fails. We recast the role of artificial intelligence in this problem: rather than predicting a binding value, which the scarce, noisy data of pre-synthetic screening cannot support, the learning layer issues a distribution-free certificate on the decision itself. We realize this as physics-constrained conformal screening, a fully in silico framework that returns not a ranked guess but a certified shortlist provably holding the best monomer. Site-directed monomer and template complexes are relaxed with the GFN2-xTB tight-binding Hamiltonian in implicit water; a competition-corrected score, $S_{\mathrm{comp}}$, discounts monomer self-association and solvation; a best-of-*k* estimator measures how far the sampling has converged; and a distribution-free conformal bound turns the result into a candidate set guaranteed, with controlled probability, to contain the optimal monomer. We prove that the corrected estimator is one-sided exact, so only the lower bound needs calibration and the guarantee requires no correction for the size of the library, and we show that a single calibration transfers between targets. Applied to two pancreatic cancer glycoepitopes, sialyl-Lewis^a^, the carbohydrate antigen 19-9 (CA 19-9) determinant, and the mucin 1 (MUC1) Tn antigen (MUC1-Tn), the framework contracts a twenty-monomer library to two and five certified candidates at ninety-percent confidence while cutting compute by 35 to 67 percent, and it identifies 3,4-ethylenedioxythiophene (EDOT) and 3-aminophenylboronic acid (3-APBA) as certified affinity optima. The same multi-seed certificate then separates the selectivity claims it can support, EDOT reading the sialylation of sialyl-Lewis^a^ and 4-aminophenylboronic acid (4-APBA) reading the Tn-glycosylation of MUC1, from the harder sialyl-Lewis^x^ regioisomer discrimination, which it flags as under-determined rather than passing off an under-sampled gap as a decisive hit, a distinction an uncertified screen cannot make. Strikingly, no point prediction model beats a trivial baseline while certification succeeds, which locates the dependable value of machine learning, for cheap physics in a low-data regime, in a guarantee rather than a guess.

## 1. Introduction

Data-driven modeling has changed how molecules and materials are discovered. Where a candidate once had to be made and measured before its worth was known, statistical models now rank thousands of possibilities in advance and send only the most promising ones to the bench [1,2]. The ambition has grown from prediction toward design, with generative and inverse methods proposing structures tuned to a target property rather than merely scoring a fixed list [3], and with hybrid schemes that let physical theory and learned models correct one another [4]. Across these advances, the recurring promise is the same, namely that computation spent early on the right molecules saves far more effort later in the laboratory.

Chemistry has absorbed this shift through learned molecular representations, message-passing networks, and force fields that reproduce quantum-mechanical energies at a fraction of their cost [5,6,7]. These tools shorten the path from a structure to an estimate of how it will behave, and they make it feasible to interrogate a library exhaustively rather than by intuition. Their reach, however, is set by a quieter question that the accuracy of a single number does not answer, which is whether that number can be trusted enough to act on.

This is where the argument of the present study begins. A point estimate carries no statement of its own reliability, and in a screening setting, a confident yet wrong ranking wastes more resources than an honest refusal to decide. Uncertainty quantification therefore moves from a refinement to a requirement [8]. Among its frameworks, conformal prediction is distinctive because it wraps any underlying model and returns set-valued outputs that carry a finite-sample, distribution-free coverage guarantee under an exchangeability assumption alone [9,10,11,12]. Its theory has matured through predictive inference for regression and resampling estimators and through conformalized quantile methods [11,13,14]; it has been extended to the covariate shift that separates training chemistry from deployment chemistry [15], and it has been used to bound the errors of machine-learned potentials before those potentials are allowed to steer a simulation [16]. The unifying lesson is that a guarantee attached to a cheap computation is often worth more than a marginally more accurate computation that offers no guarantee at all.

Few areas feel this trade-off as sharply as the design of synthetic receptors. A molecularly imprinted polymer (MIP) is an antibody mimic in which functional monomers are organized around a template and then fixed by cross-linking, so that the cured matrix keeps cavities complementary to the target in both shape and chemistry [17,18], and the resulting materials are advancing from the laboratory toward practical sensing and separation use [19]. Recognition is thus decided before polymerization at the moment that the functional monomer is chosen because that monomer sets the number, the geometry, and the strength of the contacts that the cavity will remember. Computer-assisted monomer selection has consequently become an active field, progressing from ranking monomers by a single interaction energy to broader reviews of simulation-aided and increasingly data-driven design [20,21,22]. What that literature still lacks is twofold. It commits to the top of a single computed ranking without correcting for the competing equilibria that erode affinity in a real pre-polymerization mixture, and it attaches no calibrated confidence to the choice, so the reader cannot tell how safely the shortlist can be trusted.

The clinical stakes make these omissions concrete in pancreatic ductal adenocarcinoma. Its established serum marker, carbohydrate antigen 19-9 (CA 19-9), is the sialyl-Lewis^a^ tetrasaccharide, and the aberrant mucin 1 (MUC1) carries the truncated Tn and sialyl-Tn O-glycans that accompany malignant transformation [23,24,25]. Electrochemical imprinted sensors aimed at these epitopes, including boronic acid receptors that engage the cis-diols of sialylated glycans, already reach clinically useful sensitivity [26,27,28]. Even where such sensitivity is reached, carrying it from a buffered demonstration into the protein-rich matrix of real serum remains a demanding step for the field [19]. Their weak point is selectivity against near-isomeric glycans, and the hardest case is the disease-associated sialyl-Lewis^a^ against its regioisomer sialyl-Lewis^x^, which differ only in where a single fucose is attached. A monomer that binds strongly but cannot separate these two is of no diagnostic use, so affinity and selectivity must be treated as distinct axes rather than a single score. Taken together, the gap this study addresses is a monomer screen that is at once competition-aware, able to return a shortlist with a guaranteed probability of holding the best monomer, and honest about which of its selectivity claims the available computation can actually support on this demanding isomer-discrimination task.

We close that gap with a fully in silico framework that redefines the role artificial intelligence plays in pre-synthetic screening. The conventional use of machine learning in molecular design is to predict a property value; in the low-data, high-noise regime of monomer selection that prediction is unreliable, and a confident wrong number is worse than no ranking at all. Our central move is therefore to redirect the learning layer away from prediction and toward certification, so that it issues a mathematically guaranteed statement about the decision rather than a guess about a number. This relocation of machine learning from prediction to certification is the conceptual core of the study, and it is what turns a distribution-free learning guarantee into the working engine of the screen. What makes this inversion consequential is that it changes the very question the screen is permitted to ask. A predictive model is judged by how close its number lands to an unknown truth, a standard that the scarce and noisy data of pre-synthetic screening cannot meet; a certificate is judged only by whether the shortlist it returns holds the optimum at the stated rate, a standard that needs no model of the energy landscape beyond exchangeability. To the best of our knowledge no prior approach to molecularly imprinted polymer design, and more broadly no computational receptor-screening workflow, has carried a finite-sample guarantee of this kind, and it is this move from an unattainable accuracy to an attainable confidence, far more than any single new descriptor or energy term, that we take to be the genuine advance of the work.

Concretely, the paper makes five contributions.

1. We introduce physics-constrained conformal screening, which to the best of our knowledge is the first machine learning method to attach a finite-sample, distribution-free guarantee to molecularly imprinted polymer monomer selection: it converts inexpensive tight-binding pose energies into a certified candidate set that provably retains the true optimum at a user-set confidence level, invoking no model of the energy landscape beyond exchangeability, a guarantee that no point prediction pipeline in this field currently offers.
2. We prove that the competition-corrected score inherits one-sided exactness from its estimator, so that only the lower bound must be calibrated and the retention of the optimum needs no correction for the size of the library.
3. We show that a single calibration carries across targets, letting a threshold learned on one epitope certify the other under an exchangeability assumption, so that the learned component transfers between related recognition problems.
4. We report an honest negative result that motivates the entire design: no point prediction model we tried, whether ridge regression or gradient boosting and with or without geometric descriptors, beats a trivial baseline. Far from a weakness, this is the empirical case for the whole approach, and it is precisely why the dependable machine learning contribution here is a guarantee rather than a guess.
5. We apply the framework to the two pancreatic cancer glycoepitopes above, separate affinity from selectivity, and identify 3,4-ethylenedioxythiophene (EDOT) and 3-aminophenylboronic acid (3-APBA) as certified affinity optima; the multi-seed certificate then separates the selectivity claims the library can support, EDOT reading the sialylation of sialyl-Lewis^a^ and 4-aminophenylboronic acid (4-APBA) reading the Tn-glycosylation of MUC1-Tn, from the harder sialyl-Lewis^x^ regioisomer discrimination, which it flags as under-determined rather than reporting it as a decisive hit.

Because the contribution is a method rather than the chemistry, we state it formally, so that every claim can be audited against a definition, an assumption, or a proof.

The remainder of the paper is organized as follows. Section 2 sets out the computational framework, giving the targets and the monomer library, the pose search and the tight-binding energies, the competition-corrected score, the best-of-*k* estimator, and then the machine learning certification layer itself, which is presented as Algorithm 1 and supported by four definitions, one assumption, four theorem-type results (two propositions, one lemma, and one theorem) with their proofs, and one empirical observation. Section 3 reports the results, from the reordering of the library by the competition correction through the binding modes, the selectivity analysis, the certified screening, the compute it saves, and finally the direct contrast between point prediction and certification that is the machine learning verdict of the study. Section 4 discusses what the findings mean and how they relate to the experimental literature. Section 5 states the limitations of the framework without softening them. Section 6 concludes and points to the extensions the method invites.
 **Algorithm 1** Physics -constrained conformal screening**Require:** templates *T*, monomer library, calibration group *G*, miscoverage level α, pose-budget bounds $\left[k_{\min},k_{\max}\right]$**Ensure:** a certified set $C_{q}$ per template that retains the true optimum with probability at least 1−α (Theorem 1)1: **for** each monomer *M* in the library **do**2:    evaluate the competition terms $\Delta E_{M-M}$ and $\Delta E_{M-S}$ to convergence (constants reused for every template; Lemma 1)3: **end for**4: **for** each template *T* **do**5:    **for** each monomer *M* **do**6:       k←07:       **repeat**8:         k←k+1; run one site-directed pose search returning the template-monomer interaction energy $\Delta E_{T-M}^{\left(k\right)}$; ${\hat{E}}_{k}\leftarrow \min_{j\leq k}\Delta E_{T-M}^{\left(j\right)}$9:       **until** the running minimum gains less than 0.10 kcal mol^−1^ over two successive searches with $k\geq k_{\min}$, or $k=k_{\max}$10:       ${\hat{S}}_{k}\left(M\right)\leftarrow {\hat{E}}_{k}-1/2\;\Delta E_{M-M}-1/2\;\Delta E_{M-S}$, the best-of-*k* estimate of $S_{\mathrm{comp}}$ (Equation (1); equal to $\min_{j\leq k}S^{\left(j\right)}$ by Lemma 1)11:    **end for**12:    on *G*, set $R_{M}\leftarrow \left|{\hat{S}}_{k}\left(M\right)-S_{\mathrm{comp}}^{*}\left(M\right)\right|$, with $S_{\mathrm{comp}}^{*}$ the converged reference from exhaustive sampling on *G*, and $q\leftarrow Q_{\lceil (n+1)(1-\alpha )\rceil /n}\left(\left\{R_{M}\right\}\right)$ (Equation (4))13:    $C_{q}\left(T\right)\leftarrow \left\{\;M:{\hat{S}}_{k}\left(M\right)-q\leq \min_{j}{\hat{S}}_{k}\left(j\right)\;\right\}$ (Definition 4; no multiplicity correction, Proposition 2)14: **end for**15: **if** *G* is exchangeable across templates (Assumption 1) **then** a radius *q* calibrated on one template may be reused to certify the other without recalibrating (Empirical Observation 1)16: **return** the certified set $C_{q}\left(T\right)$ for every template

## 2. Materials and Methods

**Figure 1:**
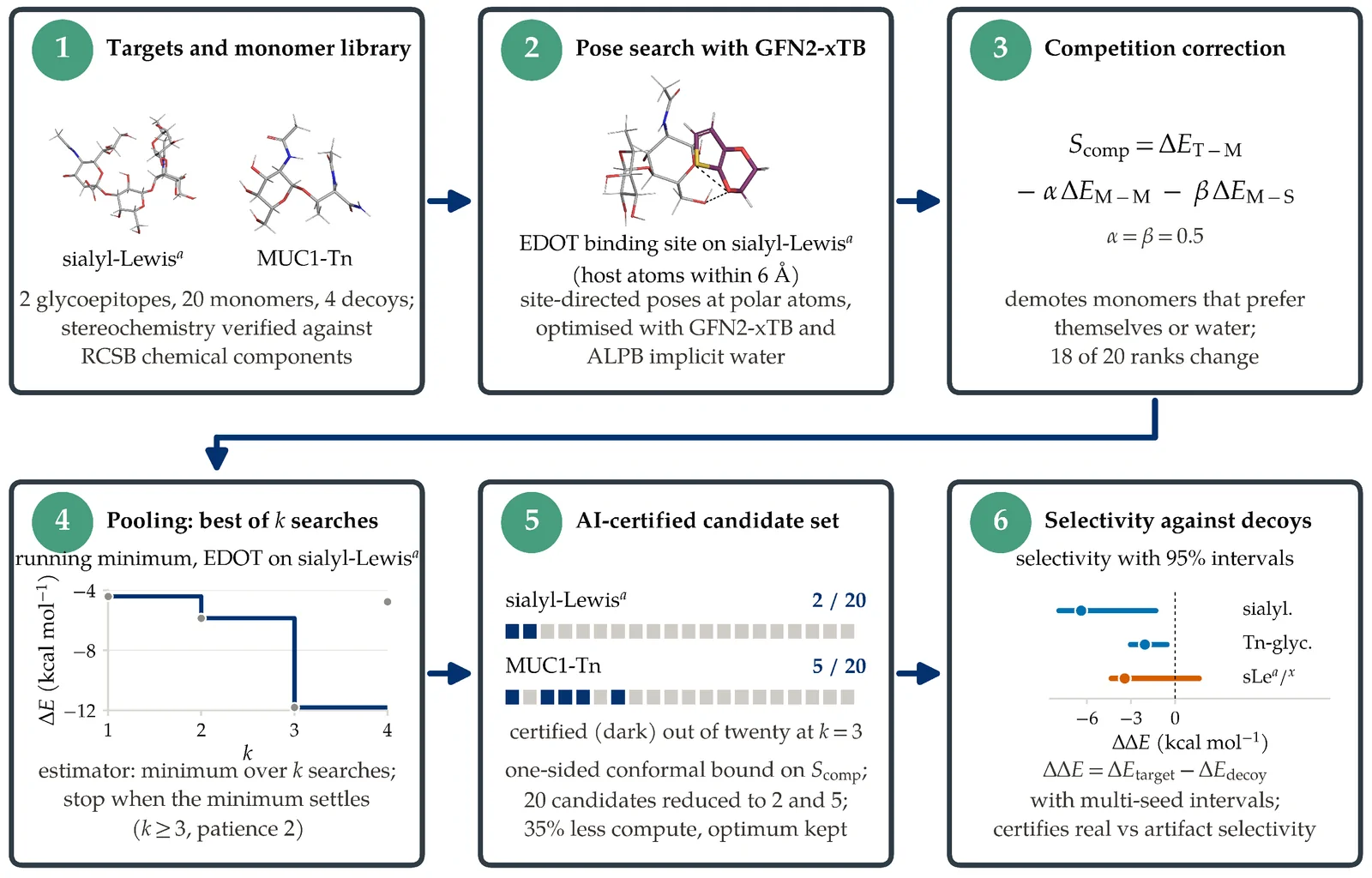
The six-stage in silico workflow. Two glycoepitope targets and a twenty-monomer library enter site-directed pose search with GFN2-xTB; the competition correction demotes monomers that prefer themselves or solvent; a best-of-*k* pooling estimator is certified by a one-sided conformal bound; and selectivity against decoy glycans is the final filter. The arrows show the left-to-right flow of the pipeline and the colors group the six stages. Every stage is rendered from the computed data rather than drawn schematically.

The framework is entirely in silico, and it is organized around a single design principle: an inexpensive but physically grounded energy is trusted only as far as a distribution-free guarantee permits, and no further. Each stage exists to serve that principle. The template and the monomer library define the search space; the site-directed pose search and the tight-binding energies supply the cheap physics; the competition correction turns a raw interaction energy into an affinity that reflects the equilibria of a real pre-polymerization mixture; the best-of-*k* estimator quantifies how much of that affinity has actually been resolved by sampling; and the conformal layer converts the whole into a certified decision. These six stages are laid out in Figure 1.

Two properties of the construction are worth stating before the details. The first is uniformity: every quantity in the paper, from the ranking of monomers to the selectivity against decoys and the certified sets, derives from one electronic structure model evaluated under one solvation treatment, so scores are directly comparable across targets and monomers and the certificate of Section 2.6 rests on a single, consistent notion of affinity. The second is reproducibility: the pose search is seeded deterministically, and the stopping rule is fixed in advance, so the reported certified sets are not an artifact of a particular random draw but a stable output of the procedure. Each subsection below pairs its computation with the formal object, a definition or a result, which makes the corresponding claim auditable.

Because the stages are arranged in the order the computation runs, Figure 1 can be read as a route through the subsections that follow. The first stage fixes the search space, the two glycoepitope targets, their four decoy glycans, and the twenty functional monomers whose structures Section 2.1 verifies against the RCSB dictionary. The second stage is the cheap physics that Section 2.2 and Section 2.3 develop, in which a monomer is placed at every polar atom of a template and the complex is relaxed with GFN2-xTB in implicit water, the inset showing one optimized EDOT pose on sialyl-Lewis^a^ together with the host atoms it contacts. The third stage is the correction of Section 2.4 that gives those energies their intended meaning, since subtracting a monomer’s self-association and its solvation demotes promiscuous binders and reorders eighteen of the twenty candidates on sialyl-Lewis^a^. The fourth stage is the sampling control of Section 2.5, which confronts pose noise head-on by pooling independent searches into a best-of-*k* minimum and halting only once that minimum has settled, the inset tracing one such running minimum for EDOT on sialyl-Lewis^a^. The fifth stage is the certification of Section 2.6 itself, where a one-sided, distribution-free bound on $S_{\mathrm{comp}}$ contracts the twenty monomers to the two and five certified candidates that provably retain the optimum while sparing a substantial share of the compute. The sixth stage is the selectivity filter of Section 2.7, which turns from affinity to discrimination by attaching a multi-seed interval to each target-versus-decoy gap ΔΔE so that a genuine selectivity can be separated from one that is merely an artifact of under-sampling. Because every inset is drawn from the actual computed output, Figure 1 is not an illustration of the method but an audit of a single pass through it.

### 2.1. Targets, Decoys, and the Monomer Library

A screen can only distinguish the chemistry it is given, so the first stage fixes that chemistry precisely. Two templates were studied, the sialyl-Lewis^a^ tetrasaccharide that constitutes the CA 19-9 epitope and a MUC1-Tn glycopeptide fragment built from N-acetylgalactosamine on threonine in its native peptide context. Four decoys probed selectivity, namely the regioisomer sialyl-Lewis^x^, the non-sialylated cores Lewis^a^ and Lewis^x^, and the bare threonine aglycone for the MUC1-Tn series. The decoy set was chosen so that each member subtracts exactly one chemical feature of its target, which turns selectivity into a controlled ablation rather than a comparison against arbitrary molecules. The regioisomer sialyl-Lewis^x^ keeps every atom of sialyl-Lewis^a^ and differs only in the linkage of a single fucose, so it isolates the hardest task of discriminating between positional isomers; the non-sialylated cores delete the sialic acid that defines the CA 19-9 determinant, so recognizing them measures whether a monomer reads the sialylation itself; and the bare threonine strips the sugar from the mucin epitope, so it reports whether binding is directed at the Tn glycan rather than at the peptide backbone. The library held twenty functional monomers, sixteen candidate receptors spanning boronic acid, amine, phenol and catechol, and heteroaromatic chemistries, together with four reference backbone monomers in common experimental use, as listed in Table 1. These chemistries were chosen to span the recognition motifs available to a synthetic receptor for carbohydrates: boronic acids that form reversible covalent esters with the cis-diols of the sugars, amines and amides for hydrogen bond donation and acceptance, phenols and catechols for combined hydrogen bonding and aromatic stacking, and electron-rich heteroaromatics for dispersion-dominated contact, while the four backbone monomers anchor the ranking to materials already in routine imprinted-polymer practice. Each template, decoy, and monomer was assigned explicit stereochemistry and a single neutral protonation state applied uniformly across the tight-binding and density-functional calculations, a modeling choice whose implications for the formally charged sialic acid are discussed in Section 5.1; every structure was checked against the Research Collaboratory for Structural Bioinformatics (RCSB) chemical component dictionary. The twenty-one stereocenters of the sialyl-Lewis glycans and the seven of the MUC1-Tn fragments were confirmed to survive every optimization, and the full structure inventory with its stereochemistry verification is given in Table S2.

### 2.2. Conformer Generation and Site-Directed Pose Sampling

With the library fixed, the geometry of each complex had to be explored. Monomer and template conformers were generated with the RDKit experimental-torsion knowledge distance geometry (ETKDG) method, version 3, and relaxed with the 94s variant of the Merck molecular force field (MMFF) [29,30]. For every template and monomer pair, candidate poses were then built by placing the monomer at each polar site of the template, meaning every hydrogen bond donor, acceptor, and diol oxygen, and by sampling rotations about the contact axis. This site-directed strategy concentrates sampling on chemically meaningful contacts instead of an undirected six-dimensional search, and it is the unit that the estimator of Section 2.5 repeats. The problem it solves is combinatorial. A blind search over the relative position and orientation of two flexible molecules spends most of its samples in sterically or chemically irrelevant arrangements, and the fraction that lands on a productive contact falls sharply as the molecules grow, so anchoring each trial at a specific polar atom removes that waste by construction and places samples only where a carbohydrate receptor can plausibly bind. Conformational flexibility is handled before docking rather than during it: ETKDG draws trial conformers from torsion angle distributions compiled from experimental crystal structures, so the starting geometries are physically reasonable rather than random, and the inexpensive MMFF94s pre-relaxation clears the worst steric clashes before any quantum-chemical work begins. The number of poses generated per contact is a budget we vary deliberately, from a coarse exploration to a dense one, so that the convergence of the ranking can itself be measured rather than assumed, a sweep reported in Section 3.2. It follows the same logic as the automated conformer-space exploration and metadynamics schemes built for fast quantum-chemical methods [31,32], and the pose relaxation and solvation settings are those recommended for robust atomistic modeling with these methods [33].

### 2.3. Tight-Binding Interaction Energies

Each pose generated in this way was then optimized and scored with the GFN2-xTB tight-binding Hamiltonian driven through the Atomic Simulation Environment with the aqueous surroundings represented by the analytical linearized Poisson–Boltzmann (ALPB) implicit solvent [34,35,36,37]. The method retains a genuine electronic structure account of polarization, hydrogen bonding, and the boron–diol interaction, including the charge-dependent dispersion correction that GFN2-xTB carries [38], at a cost low enough to make the thousands of pose evaluations, and hence the repeated sampling the calibration needs, tractable. The choice of this level of theory is deliberate, and it sits between two unsatisfactory extremes. A classical force field is fast enough for exhaustive sampling but cannot represent the partial covalency of a boronate ester or the polarization of a monomer by a charged sugar, which are the very interactions that decide carbohydrate recognition, whereas a full density-functional treatment captures that chemistry but is far too costly for the thousands of pose evaluations the calibration requires. GFN2-xTB occupies the middle ground as a self-consistent tight-binding method with charge-dependent, multipole electrostatics and an explicit account of hydrogen bonding and dispersion, so it reproduces the relevant physics at a small fraction of the density-functional cost, and its fidelity on these very complexes is checked directly against density-functional single points in Section 3.6. Solvation enters at two levels, a continuum ALPB model for the bulk aqueous environment and, through the monomer solvation surrogate term introduced below, a single explicit water molecule that stands in for the specific solvation that competes with templating. The resulting energy is defined as follows.

**Definition** **1** (Site-directed interaction energy)**.** *For a template T and a monomer M, the interaction energy of a relaxed complex is $\Delta E_{T-M}=E\left(T\cdot M\right)-E\left(T\right)-E\left(M\right)$, where the three energies are GFN2-xTB electronic energies in implicit water and each term is relaxed separately at the same level and solvation, with E(T·M) taken at the optimized pose. The monomer self-association energy $\Delta E_{M-M}=E\left(M\cdot M\right)-2E\left(M\right)$ is formed in the same way from the monomer–monomer dimer, and the monomer solvation surrogate energy $\Delta E_{M-S}=E\left(M\cdot W\right)-E\left(M\right)-E\left(W\right)$ from the complex of the monomer with a single explicit water molecule W, which serves as a local surrogate for the specific solvation that competes with templating on top of the implicit continuum. No counterpoise correction is applied, the tight-binding basis being minimal and atom-centered so that basis-set superposition error is not defined in the usual sense.*

### 2.4. The Competition-Corrected Score

A raw interaction energy over-credits monomers that bind everything strongly, because in a real pre-polymerization mixture the template competes for the monomer against the monomer’s own aggregation and against solvent. We discount those competing sinks explicitly, which gives the score that is ranked and certified throughout the paper.

**Definition** **2** (Competition-corrected score)**.** *The competition-corrected affinity of a monomer M for a template T is given by Equation (1), in which α and β weight the self-association and solvation penalties. We take α=β=1/2 throughout. A more negative $S_{\mathrm{comp}}$ marks a monomer that prefers the template over its competing equilibria. $S_{\mathrm{comp}}$ is a heuristic ranking score, not an estimate of a binding free energy: it omits entropy and explicit conformational averaging, and the weights α,β are a modeling choice whose robustness is examined in Section 3.5. Every guarantee in this work concerns the sampling error of this adopted score, not its fidelity to experiment.*

(1) $$S_{\mathrm{comp}}=\Delta E_{T-M}-\alpha \;\Delta E_{M-M}-\beta \;\Delta E_{M-S}$$

The correction is not cosmetic, and Section 3.1 shows that it reorders the library substantially. From this point onward $S_{\mathrm{comp}}$, and never the raw ΔE, is the quantity that is ranked, certified, and reported, so a single consistent definition of affinity underlies every figure and table. Because the two competition terms are computed from monomer-only optimizations that never contain the template, they are evaluated once per monomer and reused across all template-pose searches; in the implementation the pooled value of $c_{M}=\frac{1}{2}\Delta E_{M-M}+\frac{1}{2}\Delta E_{M-S}$ is numerically invariant to the pose search, agreeing across searches to within machine precision (a residual of order $10^{-16}$ kcal mol^−1^). This is the numerical counterpart of the exact cancellation that Lemma 1 establishes analytically.

### 2.5. The Best-of-k Estimator and Its Stopping Rule

A single site-directed search can miss the global pose, so the search is repeated independently and the results are pooled. The estimator and its adaptive budget are defined next, and its one-sided behavior is what later makes the certificate tight. This best-of-*k* estimate of $S_{\mathrm{comp}}$ is the single per-candidate statistic that links the physics to the certificate: it is the number the screen ranks and, unchanged, the number the conformal layer of Section 3.7 brackets, so the energy score and the guarantee are never two separate quantities but one quantity read at two stages.

**Definition** **3** (Best-of-k estimator)**.** *Let $S^{\left(1\right)},\ldots ,S^{\left(k\right)}$ be the corrected scores returned by k independent pose searches for a given template and monomer pair. The best-of-k estimator is their minimum, as in Equation (2). Sampling stops once the running minimum fails to improve by more than 0.10 kcal mol^−1^ for two consecutive searches, with k confined to the interval from $k_{\min}=3$ to $k_{\max}=10$ within a single pooling run. A small number of monomers are revisited across the screening and selectivity studies; their independent searches are pooled, so the total count reported for such a complex can exceed $k_{\max}$ by one or two searches without violating the per-run cap.*

(2) $${\hat{S}}_{k}=\min_{1\leq j\leq k}S^{\left(j\right)}$$

**Proposition** **1** (Monotone one-sided bias)**.** For each pair the estimator ${\hat{S}}_{k}$ is non-increasing in k and satisfies ${\hat{S}}_{k}\geq S^{*}$, where $S^{*}$ is the score-optimal value under the adopted model, that is, the global minimum of the competition-corrected GFN2-xTB score that unlimited pose sampling would reach. Throughout, optimum and true optimum refer to this score-optimal monomer under the adopted Hamiltonian and score, not to the experimentally best binder, which only synthesis can establish. Its bias $E\left[{\hat{S}}_{k}\right]-S^{*}$ is non-negative and non-increasing in k. Hence at small k the estimator can only under-state binding strength, that is, report a value that is too high, and never over-state it.

**Proof of Proposition** **1.** Each search returns the lowest energy among the poses it visits, so every $S^{\left(j\right)}\geq S^{*}$. The minimum of *k* such values is itself at least $S^{*}$, and it is a pointwise non-increasing function of *k* because adding searches can only lower a minimum. Taking expectations preserves both properties, and the monotonicity of the bias follows from that of the estimator. □

### 2.6. The Machine Learning Layer: Conformal Certification and Its Guarantees

The remaining step is the heart of the method. The physics of the previous subsections supplies one cheap number per monomer, and the learning layer set out here decides, with a distribution-free guarantee, which monomers may be discarded and which must be kept. The residual of the estimator, ${\hat{S}}_{k}-S^{*}$, is unknown at run time. Rather than model it, which Section 3.7 shows cannot be done reliably, we bound it with a conformal quantile and carry that bound into a candidate set that provably contains the optimum.

**Definition** **4** (Certified candidate set)**.** *Given a calibrated radius q≥0, the certified set at budget k is defined by Equation (3). It keeps every monomer whose estimated score lies within q of the current best estimate. The radius q is the conformal quantile of the absolute residuals given in Equation (4), where the empirical quantile of order ⌈(n+1)(1−α)⌉/n is taken over a calibration group of size n.*

(3) $$C_{q}=\left\{\;m:{\hat{S}}_{k}\left(m\right)-q\leq \min_{j}{\hat{S}}_{k}\left(j\right)\;\right\}$$

(4) $$q=Q_{\lceil (n+1)(1-\alpha )\rceil /n}\left(|{\hat{S}}_{k}\left(m\right)-S_{\mathrm{comp}}^{*}\left(m\right)|\right)$$

Algorithm 1 states the physics-constrained conformal screening procedure in full. Lines 1 to 11 are the cheap physics, the competition terms and the adaptive best-of-*k* pose search that produce one score per monomer; lines 12 to 16 are the machine learning layer that calibrates the conformal radius, returns a certified set, and transfers the threshold across targets. The correctness of each labeled block is settled by the formal result named against it.

**Lemma** **1** (One-sided exactness of the corrected estimator)**.** The residual sampling gain of $S_{\mathrm{comp}}$ equals that of $\Delta E_{T-M}$ exactly, because the competition terms $\Delta E_{M-M}$ and $\Delta E_{M-S}$ are converged constants that do not depend on the template-pose search. Consequently, the inequality ${\hat{S}}_{k}\geq S^{*}$ holds with probability one, so the estimator errs only on the upper side of $S^{*}$; the direction of the error is fixed, and only its magnitude below ${\hat{S}}_{k}$, the lower side of the interval around $S^{*}$, carries sampling uncertainty and so requires calibration.

**Proof of Lemma** **1.** Write $S_{\mathrm{comp}}^{\left(j\right)}=\Delta E_{T-M}^{\left(j\right)}-\frac{1}{2}\Delta E_{M-M}-\frac{1}{2}\Delta E_{M-S}$ for the *j*-th template-pose search. The subtracted competition terms are obtained from separate monomer–monomer and monomer–surrogate optimizations that do not contain the template *T*, so they carry no dependence on the index *j*; collect them into the constant $c_{M}=\frac{1}{2}\Delta E_{M-M}+\frac{1}{2}\Delta E_{M-S}$. An additive constant commutes with the minimum, so ${\hat{S}}_{k}=\min_{j}S_{\mathrm{comp}}^{\left(j\right)}=\min_{j}\Delta E_{T-M}^{\left(j\right)}-c_{M}$ and, taking the same limit for the true score, $S^{*}=\Delta E_{T-M}^{*}-c_{M}$. Subtracting cancels $c_{M}$ identically: ${\hat{S}}_{k}-S^{*}=\min_{j}\Delta E_{T-M}^{\left(j\right)}-\Delta E_{T-M}^{*}$. By Proposition 1, each search overestimates the true minimum energy, so the right-hand side is non-negative with probability one. Hence ${\hat{S}}_{k}\geq S^{*}$ always, the error is one-sided above $S^{*}$, and only its magnitude below ${\hat{S}}_{k}$ carries sampling uncertainty. □

**Assumption** **1** (Group exchangeability)**.** *The absolute residual of a test template and monomer pair is exchangeable with the absolute residuals of the calibration group G. This is the only statistical premise Algorithm 1 requires. It is much weaker than an assumption of independence of a correctly specified error model, and Section 3.7 tests it empirically across the two epitopes.*

**Theorem** **1** (Distribution-free retention of the optimum)**.** Under Assumption 1, the certified set $C_{q}$ returned by Algorithm 1 contains the true optimal monomer $m^{*}$ with the probability stated in Equation (5), for any fixed α in (0,1) and any finite calibration size n. The guarantee invokes no model of the energy landscape and no assumption on the residual distribution beyond exchangeability.

(5) $$P\left(m^{*}\in C_{q}\right)\geq 1-\alpha$$

**Proof of Theorem** **1.** The construction is split-conformal. Under exchangeability the rank of the test residual among the n+1 residuals is uniform on {1,…,n+1}, so the probability that the residual $R_{m^{*}}=\left|{\hat{S}}_{k}\left(m^{*}\right)-S^{*}\left(m^{*}\right)\right|$ does not exceed the order statistic of index ⌈(n+1)(1−α)⌉, namely *q*, is at least 1−α [10,11]. By Proposition 1 the residual is non-negative, so the event $R_{m^{*}}\leq q$ is exactly ${\hat{S}}_{k}\left(m^{*}\right)-q\leq S^{*}\left(m^{*}\right)$. Since $m^{*}$ is the global optimum, $S^{*}\left(m^{*}\right)\leq \min_{j}{\hat{S}}_{k}\left(j\right)$, and therefore ${\hat{S}}_{k}\left(m^{*}\right)-q\leq \min_{j}{\hat{S}}_{k}\left(j\right)$, which is precisely membership of $m^{*}$ in $C_{q}$ by Definition 4. Lemma 1 guarantees that the upper side carries no sampling slack, so no two-sided correction is needed. Hence $P(m^{*}\in C_{q})\geq 1-\alpha$. □

**Proposition** **2** (No multiplicity correction is required)**.** The retention guarantee of Theorem 1 needs no inflation of the radius q across the twenty monomers of the library, because it is governed by the single coverage event $|{\hat{S}}_{k}\left(m^{*}\right)-S^{*}\left(m^{*}\right)|\leq q$ for the optimum alone, and not by a simultaneous statement over all candidates.

**Proof of Proposition** **2.** Membership of a monomer in $C_{q}$ is tested against $\min_{j}{\hat{S}}_{k}\left(j\right)$, a fixed reference computed once, so retaining the designated optimum $m^{*}$ requires only that the inequality ${\hat{S}}_{k}\left(m^{*}\right)-q\leq \min_{j}{\hat{S}}_{k}\left(j\right)$ hold, which Theorem 1 shows is implied by the lone event $R_{m^{*}}\leq q$. The guarantee is thus a probability statement about one designated candidate, not about the maximum residual over the library, so the family-wise inflation that motivates Bonferroni-type corrections never enters. Enlarging the library leaves the required radius unchanged; only the reference $\min_{j}{\hat{S}}_{k}\left(j\right)$ can move, and it does so deterministically. The coverage probability of Theorem 1 therefore transfers to the retention of $m^{*}$ at level at least 1−α regardless of library size. □

**Empirical Observation** **1** (Retention at k at least three and cross-target transfer)**.** *This statement is empirical, not a consequence of the theorems above, and is labeled accordingly. For k≥3 the observed retention of the true optimum is unity across bootstrap replicates, and a radius q calibrated on one epitope still retains the optimum on the other at empirical coverage close to the nominal level. In our data, the behavior is therefore group-conditional and transfers between the two targets, within the resolution of the small calibration groups.*

**Empirical basis.** Theorem 1 guarantees only that the retention probability is at least 1−α; the stronger observed value of unity for k≥3 reflects that the residuals have collapsed below *q* once the running minimum has stabilized, as Proposition 1 predicts, and is a property of this dataset rather than a theorem. Exchangeability across epitopes is likewise an assumption rather than a proven fact, but it is borne out here: calibrating on MUC1-Tn and testing on sialyl-Lewis^a^, and the reverse, both retain the true optimum for k≥3, with empirical coverage reaching the nominal 0.90 at the operating budget k=3 and fluctuating within the noise of the small calibration group thereafter.

**Remark** **1** (Why the layer certifies rather than predicts)**.** *The guarantees above attach to a set, not to a predicted value, and that choice is deliberate. Section 3.7 shows that no regression model of the residual sampling gain beats a trivial zero-gain baseline, so a pipeline that staked its decisions on a learned property value would inherit that failure without warning. Algorithm 1 instead makes the weaker and honest claim that it can keep, with quantified confidence and without a reliable point predictor, a small set guaranteed to contain the optimum. Relocating the machine learning contribution from prediction to certification is the methodological core of the study.*

### 2.7. Selectivity Metric

Affinity alone cannot decide a receptor, so selectivity is scored separately by the differential binding energy $\Delta \Delta E=\Delta E_{\mathrm{target}}-\Delta E_{\mathrm{decoy}}$ for each monomer. A more negative value marks a stronger preference for the disease-associated target over the decoy glycan. Reporting $S_{\mathrm{comp}}$ and ΔΔE as independent axes reflects the diagnostic reality that a strong binder is useless if it cannot tell the target from its near-isomers. Because each ΔE in a selectivity gap is itself a best-of-*k* minimum over independent pose searches, we attach a finite-sample interval to every reported ΔΔE by resampling those searches: the pooled searches of the target complex and of the decoy complex are each resampled with replacement, the best-of-*k* minimum is recomputed, and the difference is formed, over twenty thousand replicates, giving a distribution-free ninety-five-percent interval. Because a resampled minimum can never lie below the deepest pose actually found, the interval is deliberately asymmetric and reports the honest risk that a decisive pose was missed at the available number of searches. Because the best-of-*k* minimum enters the selectivity gap for the target and the decoy separately, an unusually deep pose found for one complex but not matched on the other can inflate a raw gap; the resampling interval is exactly what exposes such a gap as under-determined, so a selectivity is certified here only when the interval, and not merely the point estimate, excludes zero.

### 2.8. Software and Reproducibility

The calculations used GFN2-xTB and GFN1-xTB as implemented in tblite 0.6.0, driven through the Atomic Simulation Environment 3.29.0 [37], with RDKit 2026.09.1 for cheminformatics, scikit-learn 1.9.1 [39] for the regression baselines and the conformal calibration, and PyMOL 3.2 for structure rendering; the deprotonated-state re-screen used the standalone xtb program 6.7.1. The density-functional cross-check of Section 3.6 used the Perdew–Burke–Ernzerhof (PBE) functional with the D3(BJ) dispersion correction [38,40] and a def2-SVP basis, evaluated as single points on the tight-binding geometries. Every tight-binding score derives from one energy model, and all random seeds are fixed so the certified sets are reproducible.

## 3. Results

The framework of Section 2 is now applied to the two glycoepitopes, and the results are arranged to follow the logic of a decision rather than a catalog of measurements. We begin with the effect of the competition correction, since it changes which monomers deserve attention at all, and then examine the geometries behind the leading scores to confirm that the numbers correspond to chemically sensible recognition. From there the account turns to selectivity, the property on which a diagnostic receptor ultimately stands or falls, and to the sharper question of which selectivity claims survive a quantified sampling uncertainty. We next probe the two modeling choices that could most plausibly undermine the ranking, the competition weights and the electronic structure method itself, before the certification layer draws the strands together, allocates computation adaptively, and is weighed directly against point prediction in the machine learning verdict that closes the section. Throughout, every reported quantity is a certified output of the single pipeline of Section 2, so the results read as one continuous argument from cheap physics to a guaranteed decision rather than as a series of separate tests.

### 3.1. The Competition Correction Reorders the Library

**Figure 2:**
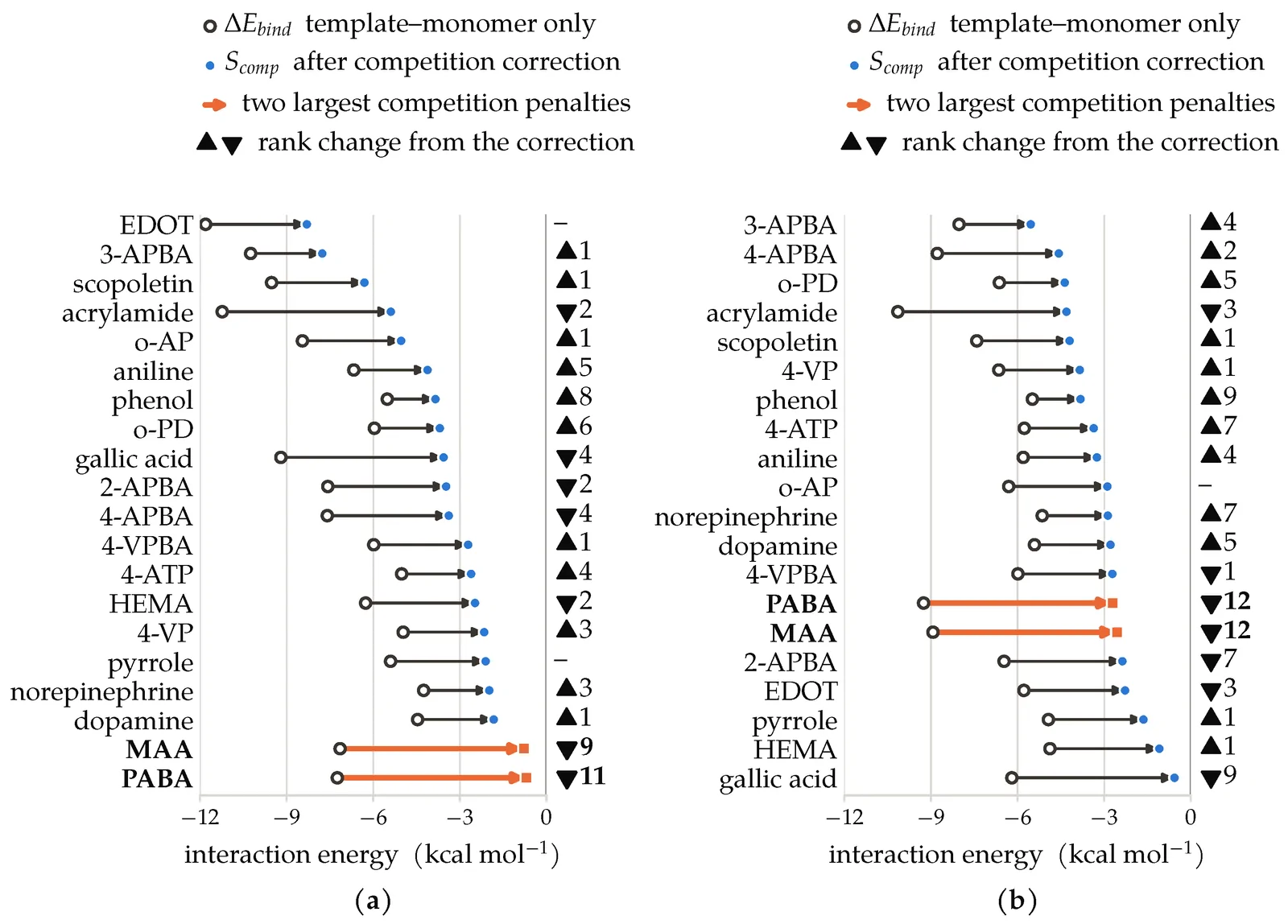
The effect of the competition correction for (**a**) sialyl-Lewis^a^ and (**b**) MUC1-Tn. In each panel the open circles give the raw interaction energy $\Delta E_{\mathrm{bind}}$ (template–monomer only) and the blue filled circles the competition-corrected score $S_{\mathrm{comp}}$; the orange segments mark the two monomers with the largest competition penalties, and the black up and down triangles with the adjacent number give each monomer’s rank change from the correction. Ranking by ΔE and by $S_{\mathrm{comp}}$ reorders the library, demoting self-associating monomers such as acrylamide and promoting directionally binding monomers; all twenty monomers change rank on at least one target, eighteen of them on sialyl-Lewis^a^ and nineteen on MUC1-Tn.

Ranking the twenty monomers by the raw $\Delta E_{T-M}$ and by $S_{\mathrm{comp}}$ produces materially different shortlists, and all twenty monomers change rank on at least one target, eighteen of them on sialyl-Lewis^a^ and nineteen on MUC1-Tn, as Figure 2 shows. The clearest casualty is acrylamide. It binds both templates strongly in isolation, ranking second on sialyl-Lewis^a^ and first on MUC1-Tn by raw energy, yet it self-associates so strongly, with $\Delta E_{M-M}$ near −8.9 kcal mol^−1^, that the correction sends it to fourth on both. In the opposite direction, EDOT and 3-APBA engage the templates through directional contacts rather than aggregation and rise to the top of the corrected ranking. Table 2 lists the five highest-ranked monomers per epitope by $S_{\mathrm{comp}}$, with EDOT the corrected optimum for sialyl-Lewis^a^ at −8.30 kcal mol^−1^ and 3-APBA the optimum for MUC1-Tn at −5.54 kcal mol^−1^; the competition-corrected scores for all template and monomer pairs are tabulated in Table S1.

### 3.2. Convergence of the Estimator and Stoichiometry

**Figure 3:**
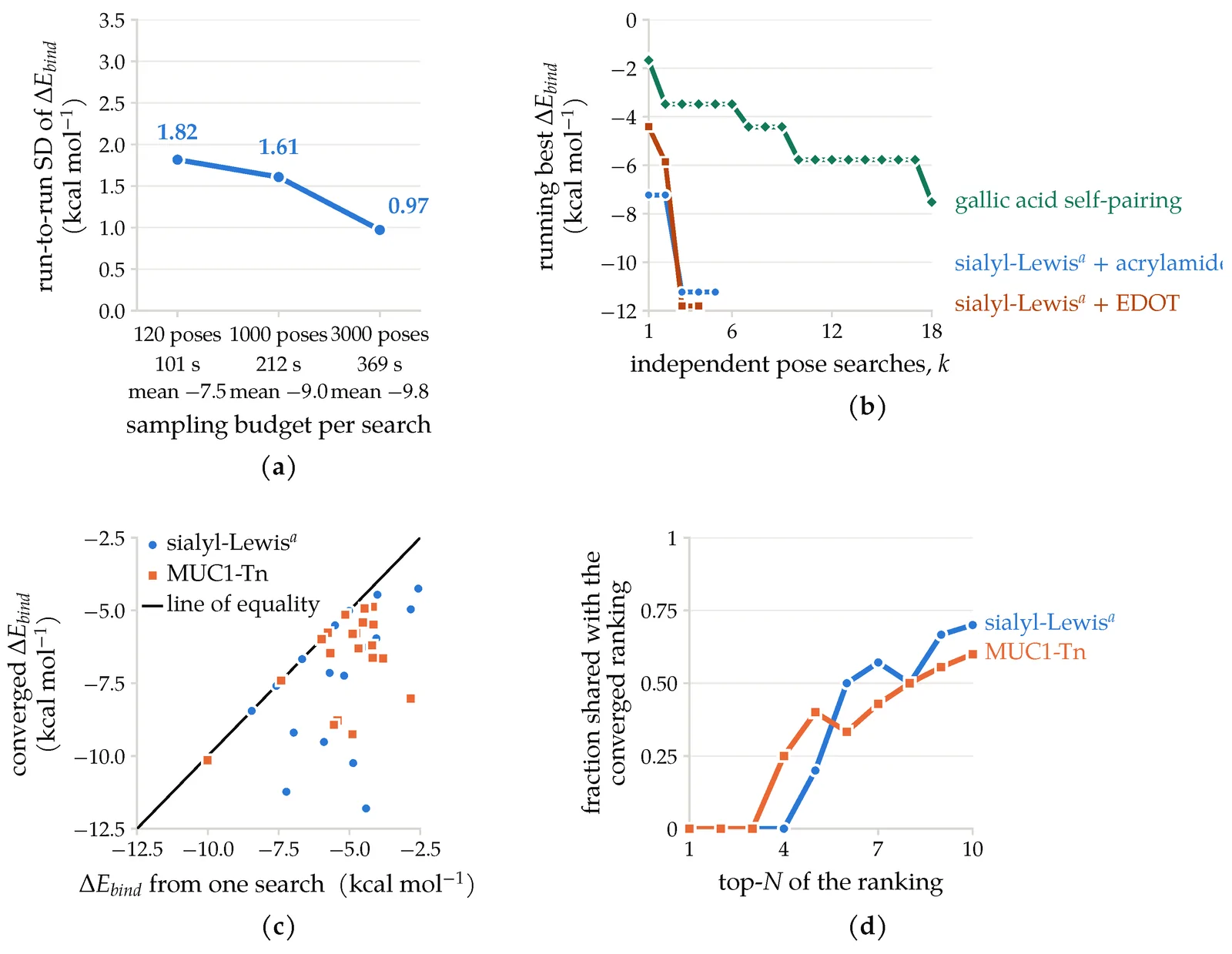
Convergence of the best-of-*k* estimator. (**a**) The run-to-run standard deviation of the binding energy $\Delta E_{\mathrm{bind}}$ falls as the sampling budget per search increases (120, 1000, and 3000 poses; 101, 212, and 369 s mean cost), from 1.82 to 0.97 kcal mol^−1^. (**b**) The running best $\Delta E_{\mathrm{bind}}$ against the number of independent pose searches *k* for three representative template–monomer pairs. (**c**) The converged $\Delta E_{\mathrm{bind}}$ against the value from a single search for sialyl-Lewis^a^ (blue circles) and MUC1-Tn (orange squares), with the line of equality. (**d**) The fraction of the top-*N* ranking shared with the converged ranking against *N*. The mean and worst-case residual sampling errors both decrease monotonically with *k* and stabilize by k=3, which the adaptive stopping rule exploits.

For the ranking to be trustworthy, the estimator must have converged, and it does so quickly. The mean residual sampling error falls from 1.67 kcal mol^−1^ at k=1 to 0.33 at k=3 and 0.15 at k=4, while the worst-pair error settles near 2.1 kcal mol^−1^ by k=3, as Figure 3 shows. This behavior is what justifies the stopping rule of Definition 3, which halts most pairs at k=3 or k=4. A budget analysis in Table 3 makes the trade-off explicit, since raising the pose budget from 120 to 3000 lowers the mean ensemble energy from −7.52 to −9.80 kcal mol^−1^ and halves the within-pair spread. A stoichiometric analysis of the leading monomers, given in Figure S1, confirms that the reported contacts correspond to one-to-one association rather than to incidental stacking. The energies reported here and in Table 2 are best-of-*k* point estimates, and a few deep poses are recovered only occasionally, so individual values carry the pose-sampling uncertainty that the certification layer quantifies in Section 3.4 and Section 3.7; this is exactly why the framework delivers a certified set rather than a point ranking.

### 3.3. Binding Modes

**Figure 4:**
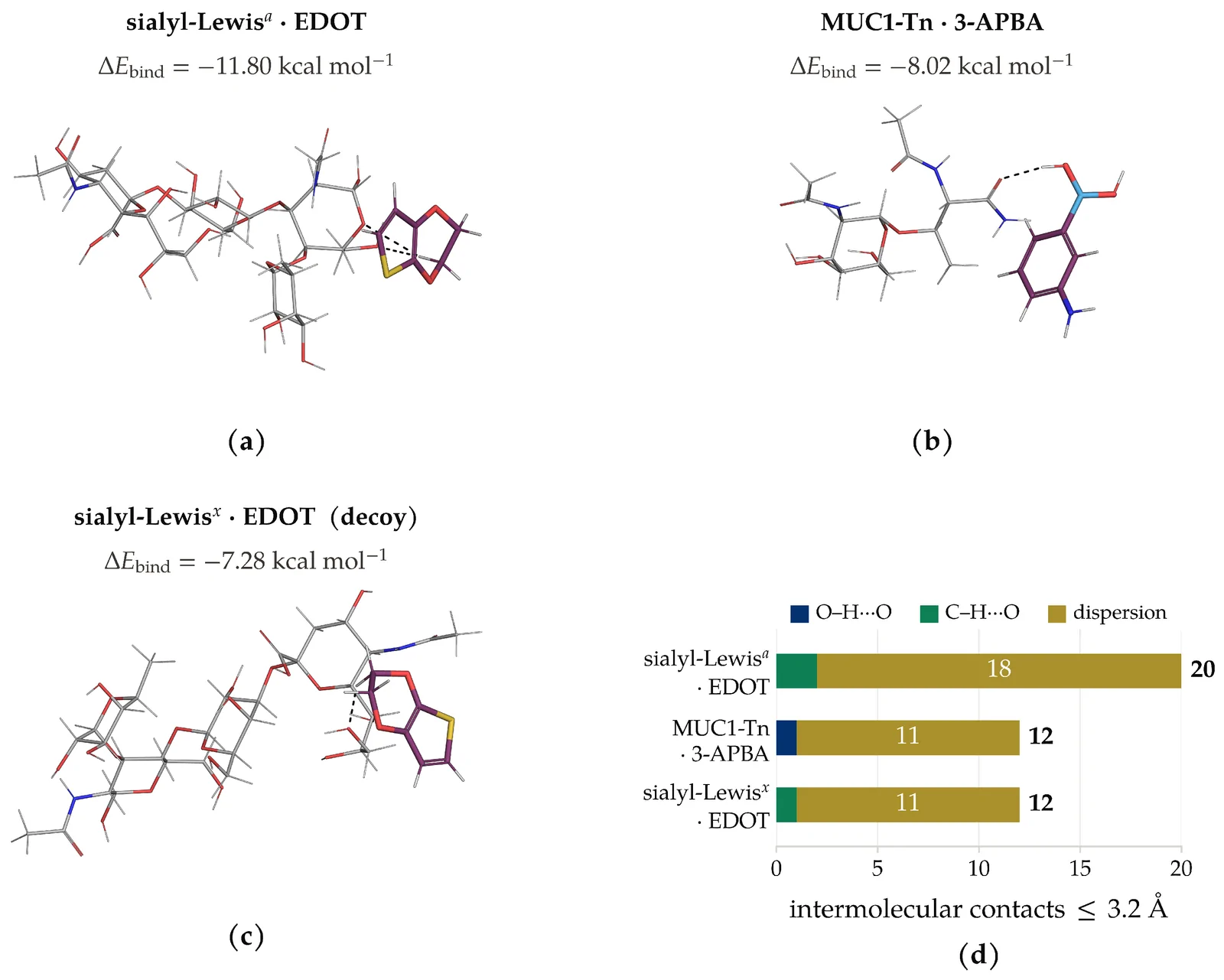
The certified binding modes. (**a**–**c**) show EDOT on sialyl-Lewis^a^, 3-APBA on MUC1-Tn, and EDOT on the sialyl-Lewis^x^ decoy, drawn at a common scale with explicit bond orders; atoms follow the standard element color scheme (carbon gray, oxygen red, nitrogen blue, boron pink, sulfur yellow) and dashed lines mark the key hydrogen-bond and polar contacts. (**d**) gives the contact inventory within 6 Å of the monomer. All geometries passed the structural checks.

Beyond the numbers, the certified optima adopt chemically sensible geometries, and Figure 4 shows them at their best poses so that the scores can be read against the physical contacts that produce them. In Figure 4a EDOT lies flat across the sialyl-Lewis^a^ face in close van der Waals contact, a dispersion-dominated association that is spread over the sugar surface rather than anchored at a single group and that forms no dedicated hydrogen bond. This diffuse, non-directional mode is exactly what one expects of a monomer that cannot tell the two sialyl-Lewis isomers apart, and it is the geometric counterpart of the weak, under-determined isomer gap that Section 3.4 reports. Figure 4b shows the opposite kind of binding. The boronic acid monomer 3-APBA engages the MUC1-Tn N-acetylgalactosamine through a specific boronate-and-amine motif poised over the cis-diol, a directional, close-fitting contact that pins the monomer to the one chemical feature distinguishing the Tn sugar, and it is this geometry that underlies the monomer’s certified selectivity. Figure 4c places EDOT on the sialyl-Lewis^x^ decoy for direct comparison, where it adopts a nearly identical flat stacking and gains no new specific contact, which makes visible why the isomer discrimination is not robust: the monomer sees the two regioisomers through almost the same interface. Figure 4d sets the two modes against each other through the inventory of host atoms within 6 Å of the monomer, making explicit that the boronate recognition rests on a few close polar contacts whereas the EDOT association is distributed across many weaker van der Waals neighbors. Every reported complex was audited for geometric fidelity: no bond deviated from its expected length, no non-bonded pair collapsed to a covalent distance, all stereocenters were preserved, and every aromatic ring stayed planar to within a root-mean-square deviation of 0.01 Å, with bond orders assigned from the molecular topology so that aromatic and multiple bonds render explicitly rather than as uniform sticks.

### 3.4. Selectivity, and Which Selectivity Claims Survive Certification

**Figure 5:**
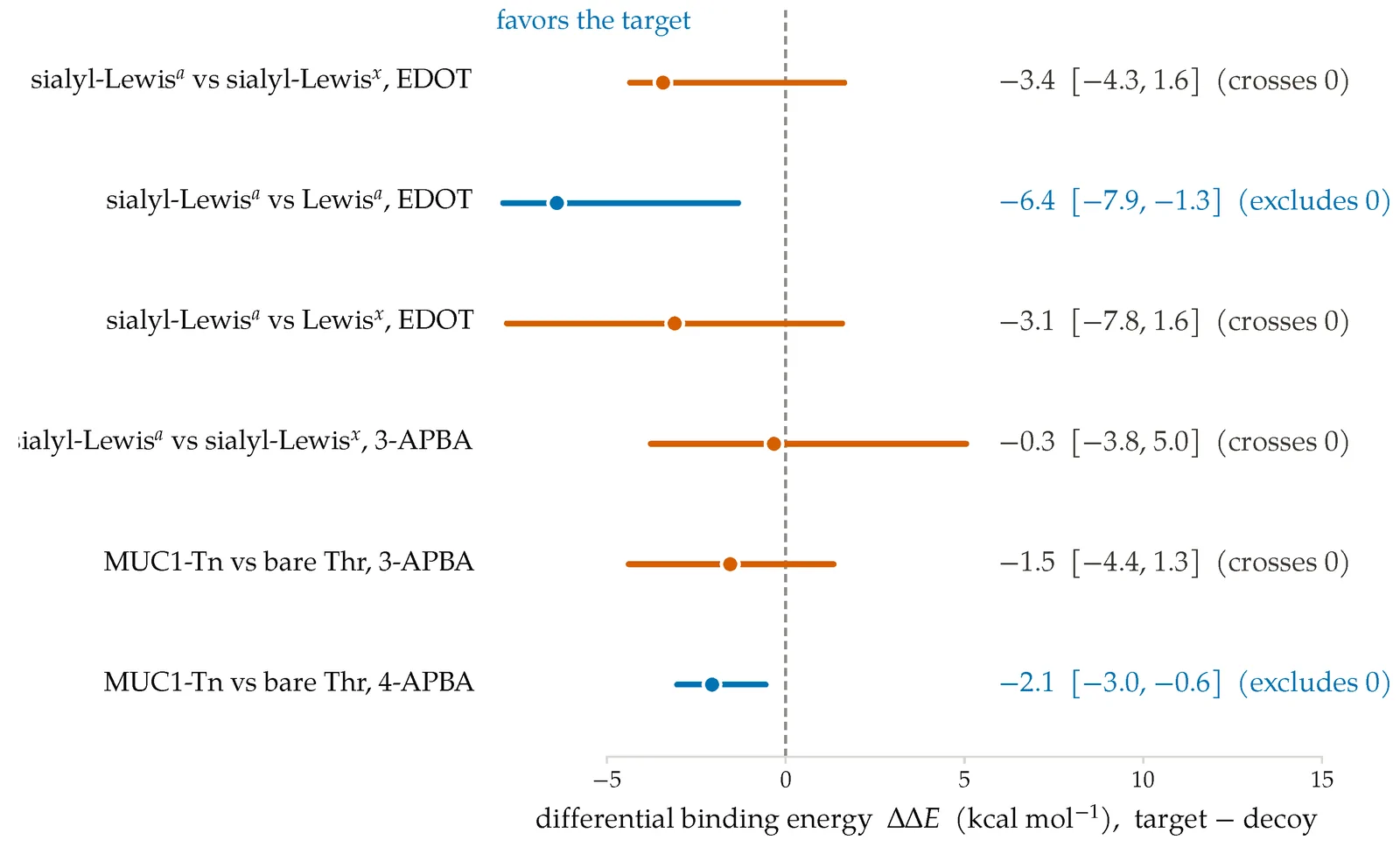
Selectivity with quantified sampling uncertainty. Each differential binding energy $\Delta \Delta E=\Delta E_{\mathrm{target}}-\Delta E_{\mathrm{decoy}}$ is shown with its distribution-free ninety-five-percent multi-seed interval. Intervals that lie entirely below zero (blue) mark selectivity that survives certification, namely EDOT reading the sialylation of sialyl-Lewis^a^ and 4-APBA reading the Tn-glycosylation of MUC1-Tn; intervals that cross zero (orange), including the sialyl-Lewis^a^ against sialyl-Lewis^x^ regioisomer gap, are flagged as under-determined at the available pose budget.

Selectivity is where the certification layer earns its place, because a differential binding energy is only as trustworthy as the pose search behind each of its two terms. We therefore attach to every ΔΔE the distribution-free ninety-five-percent interval of Section 2.7, obtained by resampling the independent pose searches, and judge a selectivity claim robust only when that interval excludes zero. Figure 5 reports the gaps in this footing, and the result is a clean separation between claims that survive and claims that do not.

Two recognition events are robust. EDOT distinguishes the sialylated sialyl-Lewis^a^ epitope from its non-sialylated Lewis^a^ core by ΔΔE=−6.4 kcal mol^−1^ (ninety-five-percent interval excluding zero), so the sialic-acid substituent that defines the CA 19-9 determinant is read directly. On the mucin epitope the boronic acid monomer 4-APBA distinguishes the Tn-glycosylated threonine from the bare threonine by −2.1 kcal mol^−1^ (interval [−3.0,−0.6]), the boron–diol recognition of the Tn sugar that the chemistry predicts. These are the selectivity results the framework certifies. Both boronic acid claims rest on the production multi-seed pools rather than a dedicated equal-depth re-sample of the kind run for the EDOT isomer pair, so their point magnitudes carry the same best-of-*k* sampling caveat; what certifies them is that the resampling interval, not the point estimate alone, excludes zero.

The harder claim does not survive, and the certification layer is what exposes it. Discriminating sialyl-Lewis^a^ from its regioisomer sialyl-Lewis^x^, which differ only in the linkage of a single fucose, is the diagnostically decisive task, and a single-shot best-of-*k* screen appears to solve it: it returns ΔΔE=−4.5 kcal mol^−1^ for EDOT in favor of the disease-associated isomer. That gap, however, rests on one unusually deep sialyl-Lewis^a^ pose that carries no hydrogen bond and was recovered only once in eighteen independent searches pooled for this complex. Carried into the multi-seed certificate of Figure 5, that single pose still leaves a best-of-*k* point estimate of −3.4 kcal mol^−1^, but its ninety-five-percent interval, [−4.3,+1.6], already spans zero, so the apparent preference cannot be separated from none even before the search effort is equalized. When both isomer complexes are then searched with equal and sufficient effort, their best-of-*k* energies become statistically indistinguishable, −8.4 against −8.4 kcal mol^−1^, confirming directly that the apparent gap is an artifact of unequal sampling depth. Quantifying the required budget directly from deep independent sampling of the two isomers (fourteen and thirteen searches for the decisive EDOT pair), the best-of-*k* energies become statistically indistinguishable only once *k* reaches about 11 to 13: the running best-of-*k* stabilizes to within 0.1 kcal mol^−1^ by k=11 for sialyl-Lewis^a^ and k=8 for sialyl-Lewis^x^, and at the full budget the gap is −0.02 kcal mol^−1^ with a distribution-free ninety-five-percent interval of [−0.94,+1.74] kcal mol^−1^ that spans zero. Ordinary monomer-template pairs converge by k=3 to 4, as the convergence study of Figure 3 records, so resolving this regioisomer discrimination would require a budget of order k≥11, roughly three to four times larger, and Figure S2 traces this isomer convergence. The apparent regioisomer selectivity is therefore an artifact of under-sampling, not a property of the monomer, and the multi-seed certificate flags it as under-determined where an uncertified screen would have reported it as a decisive hit. Exposing this distinction, between a selectivity that is real and one that is a sampling accident, is precisely the diagnostic the method is built to provide. The full differential-binding matrix with intervals is given in Table S3.

### 3.5. Sensitivity to the Competition Weights

**Figure 6:**
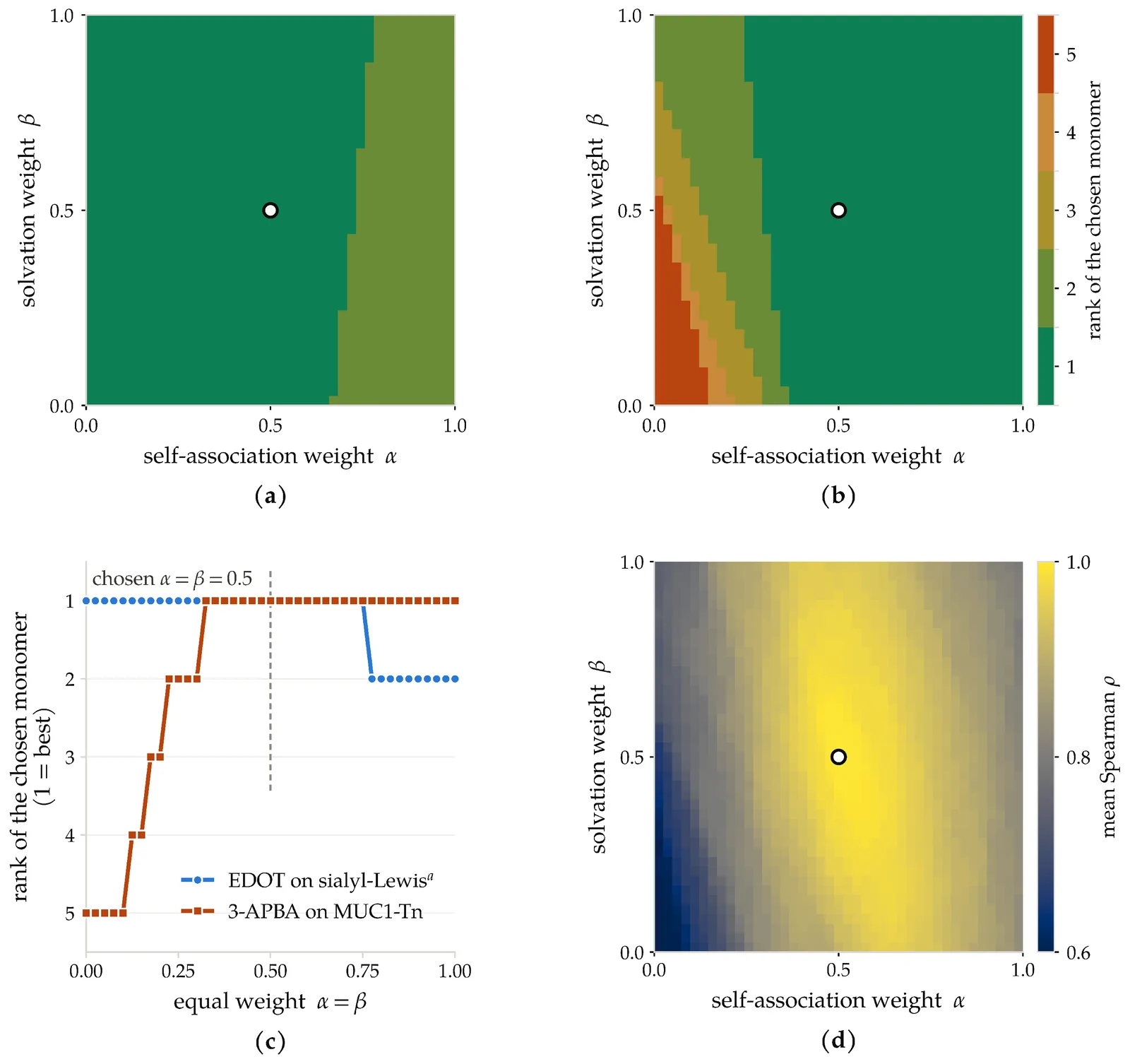
Sensitivity of the ranking to the competition weights α and β, where rank one denotes the best-scoring monomer. (**a**) maps the rank of EDOT on sialyl-Lewis^a^ and (**b**) the rank of 3-APBA on MUC1-Tn over the (α,β) unit square, sharing the discrete rank color scale shown at the right of (**b**); the open circle marks the chosen α=β=1/2. (**c**) follows the two optima along the equal-weight diagonal, with rank increasing downward; (**d**) maps the mean Spearman correlation of the full ranking against the α=β=1/2 baseline, so that bright regions denote weightings that leave the ordering essentially unchanged.

The competition weights α and β were fixed at 1/2, and it is fair to ask how far the conclusions depend on that choice. We swept both weights over the unit square and recorded, at every setting, the rank that each chosen optimum would receive, with the result summarized in Figure 6. For sialyl-Lewis^a^ the optimum EDOT is ranked first over 73 percent of the grid and never falls below second, so its selection is essentially independent of the weighting, as Figure 6a shows. For MUC1-Tn the optimum 3-APBA is ranked first over 70 percent of the grid and always stays within the top five; it loses the leading position only in the low-weight corner where the competition penalties are switched off, which is precisely the regime the correction exists to avoid, as Figure 6b shows. Along the equal-weight diagonal, 3-APBA becomes the optimum once α=β exceeds about 0.33 and stays so thereafter, while EDOT is optimal up to about 0.75, as Figure 6c shows. The full ranking is stable near the chosen point, with a mean Spearman correlation above 0.9 against the α=β=1/2 baseline across most of the square, degrading only in the extreme corner where competition is ignored, as Figure 6d shows. Read together, the four views say more than that the result is stable. The two optima occupy wide, well-separated basins of the weight plane rather than winning by a narrow margin at one setting, so the chosen α=β=1/2 sits comfortably in the interior of each, far from the boundary where the ranking would flip. That the near-unity Spearman correlation holds for the full ordering, not merely for the top-ranked monomer, shows the correction reshapes the library in a smooth and reproducible way rather than reshuffling it, and it isolates the low-weight corner, where the penalties are effectively switched off, as the only regime into which the conclusions should not be read. The central conclusions are therefore robust to the one modeling parameter that the physics does not fix, and the single degree of freedom the score introduces is shown to be a mild one.

### 3.6. Cross-Method Validation of the Energy Model

**Figure 7:**
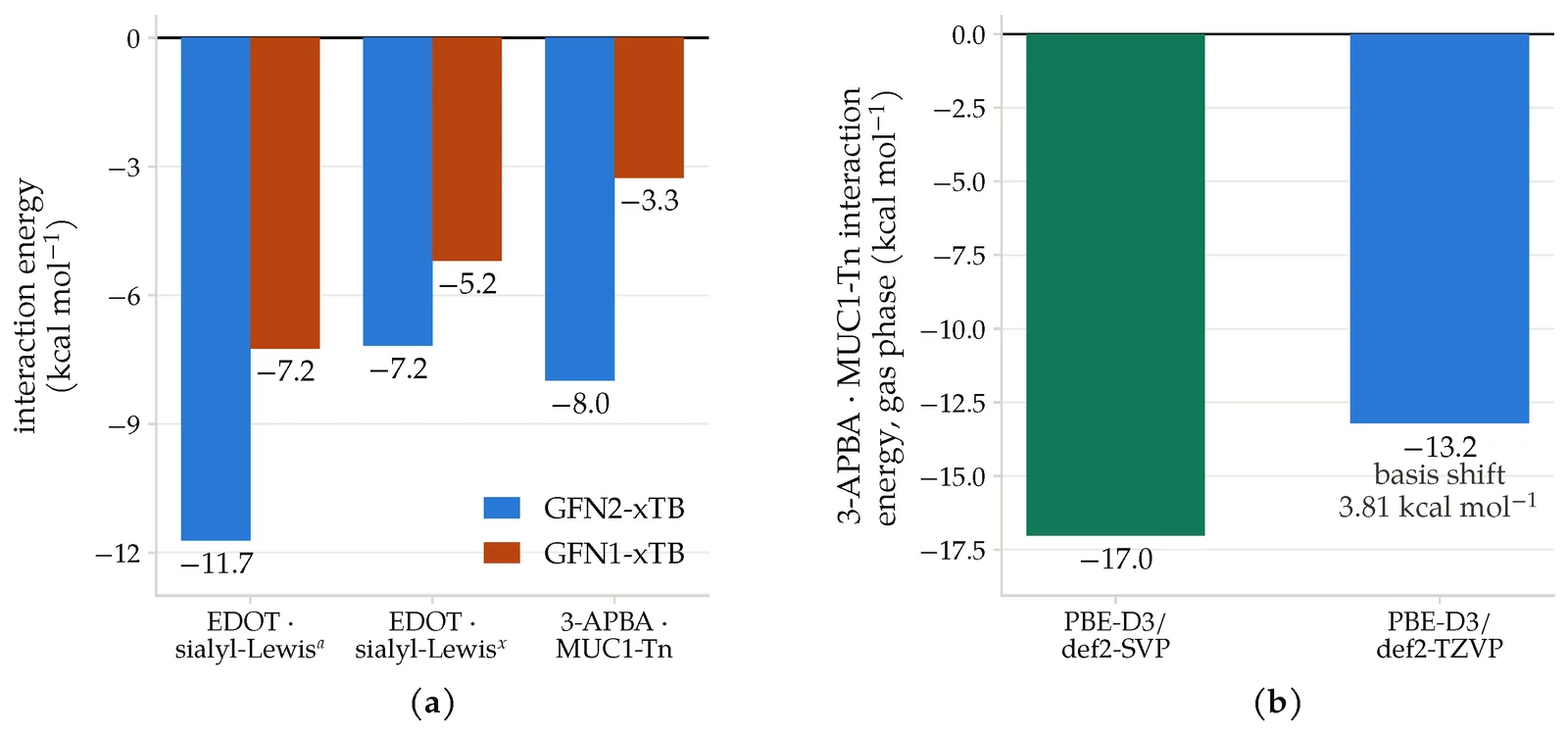
Cross-method validation of the energy model at fixed geometry. (**a**) compares the interaction energies of the three key complexes under two tight-binding parametrizations, GFN2-xTB and GFN1-xTB, at their saved best-pose geometries and under the same implicit solvation, which preserve their ordering; the sialyl-Lewis^a^ bar is the rare deep pose discussed in Section 3.4 and is shown here only to compare methods at a fixed geometry, not as evidence of selectivity; (**b**) shows the basis convergence of the density-functional check on the boron–diol recognition of the Tn sugar: the gas-phase PBE-D3(BJ) interaction energy is 3.8 kcal mol^−1^ less bound in the larger def2-TZVP basis than in def2-SVP, as basis-set superposition error is removed, yet remains strongly favorable. The check validates the energy model, not any selectivity, since every method is evaluated at the same geometry.

The certification layer isolates the uncertainty that comes from pose sampling, but a second question is whether the underlying tight-binding energies are themselves trustworthy at a fixed geometry. This is a property of the Hamiltonian and the basis, not of the sampling, and we test it directly. Re-evaluating the GFN2-optimized geometries with GFN1-xTB, a different tight-binding parametrization under the same implicit solvation, preserves the ordering of interaction energies for all three key complexes, although GFN1-xTB binds systematically more weakly, as Figure 7a,b show. A first-principles check places the boron–diol recognition of the Tn sugar on a firmer footing: at the gas-phase PBE-D3(BJ) level the interaction energy is −13.2 kcal mol^−1^ in the larger def2-TZVP basis, some 3.8 kcal mol^−1^ less bound than in def2-SVP once basis-set superposition error is removed, but still strongly favorable. That absolute energies are this sensitive to the basis is exactly why selectivity is judged throughout from consistently computed energy differences rather than absolute values, and why the reliability of a selectivity is governed by the pose sampling of Section 3.4, not by the level of theory. Taken together the two tight-binding parametrizations and the density-functional single points agree on the interaction energies at a given geometry, so the electronic structure description is not the weak link. It is worth stating plainly what this does and does not establish: it validates the energies the screen is built on, but it cannot rescue a selectivity that rests on an under-sampled pose, because every method here is evaluated at the same geometry. The reliability of a selectivity is settled by Section 3.4, the fidelity of the energy model by this section, and the two must not be confused. These are two distinct kinds of uncertainty, and the framework addresses them with two distinct instruments: the agreement among GFN2-xTB, GFN1-xTB, and the density-functional single points bounds the epistemic, or model, uncertainty in the energy at a fixed geometry, whereas the conformal certificate bounds the sampling uncertainty in locating the pose, so a reader can see at a glance which guarantee speaks to which risk.

### 3.7. Machine Learning That Certifies Rather than Predicts

**Figure 8:**
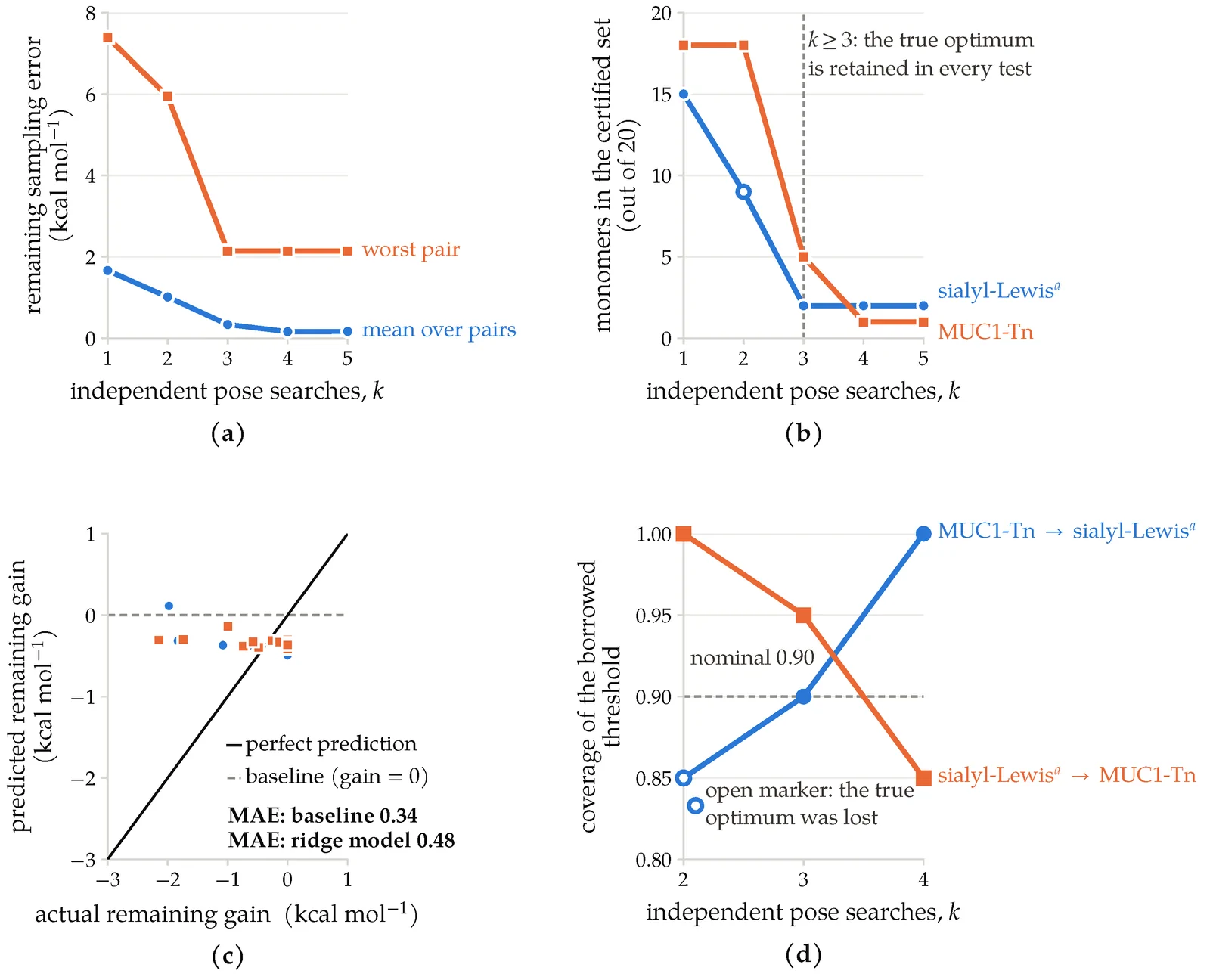
What can and cannot be certified after *k* cheap searches. (**a**) shows the residual sampling error decaying with *k*. (**b**) shows the certified set contracting to two and five monomers while retaining the true optimum for k≥3; (**c**) shows that a learned regressor does not beat the zero-gain baseline, with blue circles for sialyl-Lewis^a^ and orange squares for MUC1-Tn; (**d**) shows a threshold calibrated on one epitope transferring to the other with the true optimum retained and empirical coverage close to the nominal level.

This subsection is where the machine learning layer does its work, and where its role is deliberately not the one usually assigned to it. Rather than train a model to predict a binding property and then act on the prediction, the learning layer is asked only to decide, with a distribution-free guarantee, which monomers may be discarded and which must be kept. The certification brings the preceding strands together. At the ninety-percent level, with α=0.10 and k=3, the conformal procedure contracts the twenty-monomer library to a certified set of two candidates for sialyl-Lewis^a^, namely EDOT and 3-APBA, and five for MUC1-Tn, namely 3-APBA, acrylamide, phenol, o-phenylenediamine, and scopoletin, while retaining the true optimum in every case, as Figure 8a,b show. This success stands in deliberate contrast to point prediction. A leave-one-out ridge regressor trained on molecular and geometric descriptors to predict the residual sampling gain reaches a mean absolute error (MAE) of 0.48 kcal mol^−1^, which is worse than the trivial baseline that predicts zero gain, at 0.34 kcal mol^−1^. This baseline is the constant predictor that takes the cheap best-of-*k* energy at face value, that is, a predicted residual gain of zero; it is not random selection or frequency-based guessing. It is moreover near-optimal: a baseline that instead predicts the mean residual has a larger error, 0.45 kcal mol^−1^, because the gains are small and right-skewed with a median close to zero, so the zero-gain bar is not set artificially low. The ridge model selected its regularization by cross-validation, and the gradient-boosted model used standard tuned settings, so the failure to beat the baseline reflects the low-data regime rather than a weak implementation. Gradient boosting and the addition of geometry features do not help, as Table S4 and Figure 8c record. To a learned model the residual is effectively irreducible noise, yet the conformal quantile bounds it exactly. The threshold is also portable, since a radius calibrated on one epitope transfers to the other while retaining the true optimum, with empirical coverage reaching the nominal 0.90 at the operating budget k=3, as Figure 8d shows, matching Empirical Observation 1. The lesson for a data-scarce design problem is pointed. The learning layer earns its place not by predicting the quantity of interest, which the regression experiments here show it cannot do, but by certifying, with a finite-sample guarantee, that the cheap physics has already located the answer; the intelligence of the procedure lies in knowing when to stop computing, not in guessing a number it has too little data to learn.

Because a conformal guarantee is only as honest as its calibration, we stress-tested the exchangeability assumption directly rather than assuming it. In a strict within-target leave-one-monomer-out protocol, where the quantile for each held-out monomer is calibrated only on the remaining nineteen of the same epitope, the empirical coverage equals the nominal 0.90 for both epitopes and for the pooled set of forty residuals at k=3, confirming that the certified sets are not an artifact of a monomer calibrating its own radius. The residuals also show no dependence on molecular size, the Spearman correlation between the residual magnitude and the atom count being −0.08 (*p* =0.62). They do, however, depend on the monomer family: the sixteen electropolymerizable monomers carry a mean residual of 0.19 kcal mol^−1^, against 0.88 kcal mol^−1^ for the four classical acrylic monomers, which have rougher pose landscapes (Mann-Whitney *p* <0.001). We therefore report the subgroup coverage explicitly. Under the pooled calibration (n=40, radius 1.74 kcal mol^−1^ at ninety percent), the empirical coverage is 0.97 (31 of 32) for the electropolymerizable set but 0.75 (6 of 8) for the acrylic subset; under a strict leave-one-class-out calibration, using only the electropolymerizable family, acrylic coverage falls further to 0.625 (5 of 8). This is the expected mild anticonservatism of pooling a minority class with heavier tails. Because the minority family holds only four members, its own ninety-percent quantile is not finitely estimable. We therefore pool the families for calibration and flag, in Section 5, the mild anticonservatism this introduces across the family boundary; within a family, and across size, the guarantee holds at its nominal level. Class-conditional bounds would remove this residual anticonservatism once a library is balanced enough for each family to support its own quantile.

### 3.8. Certified Budget Allocation

**Figure 9:**
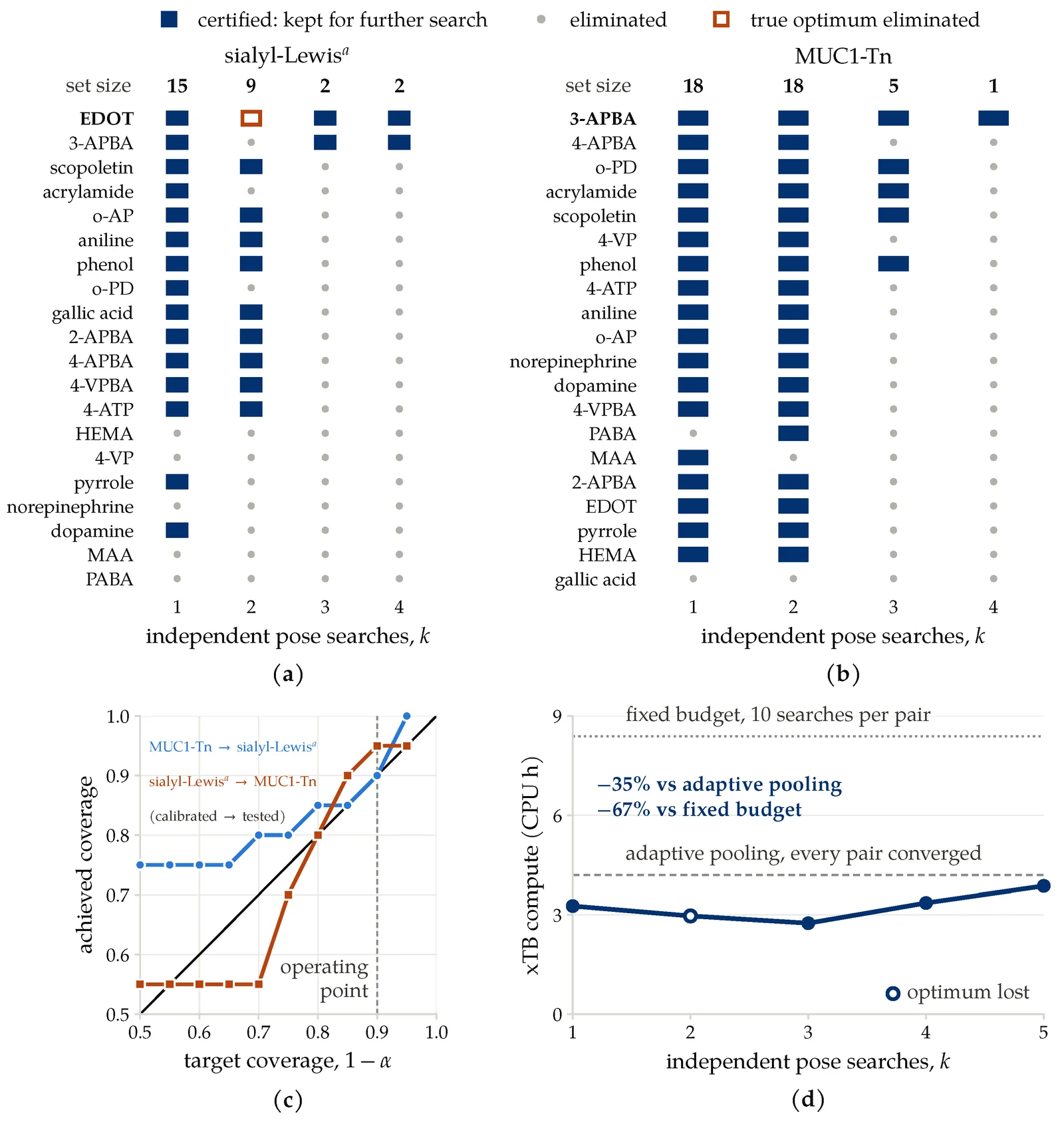
Certified screening and budget allocation. (**a**,**b**) are elimination maps that show monomers being certified out of the running as *k* grows, one per epitope; (**c**) shows the calibration of the conformal radius; (**d**) shows the computational cost of the certified strategy against undirected pooling and a fixed high-budget sweep, giving savings of thirty-five to sixty-seven percent at equal retention.

Because the certified set is settled by the conformal quantile rather than by exhaustive search, computational resources can be allocated adaptively, as Figure 9 shows. At α=0.10 and k=3 the framework reaches the final certified sets using 2.75 central processing unit (CPU) hours of tight-binding evaluation, against 4.20 CPU-hours for undirected best-of-*k* pooling and 8.39 CPU-hours for a fixed high-budget sweep. These are reductions of thirty-five and sixty-seven percent, with the true optimum retained in every certified configuration. The contribution of the learning layer is therefore quantitative and auditable, since it is the machinery that decides, with a guarantee, when enough computation has been done.

### 3.9. The Machine Learning Verdict: Certify, Do Not Predict

**Figure 10:**
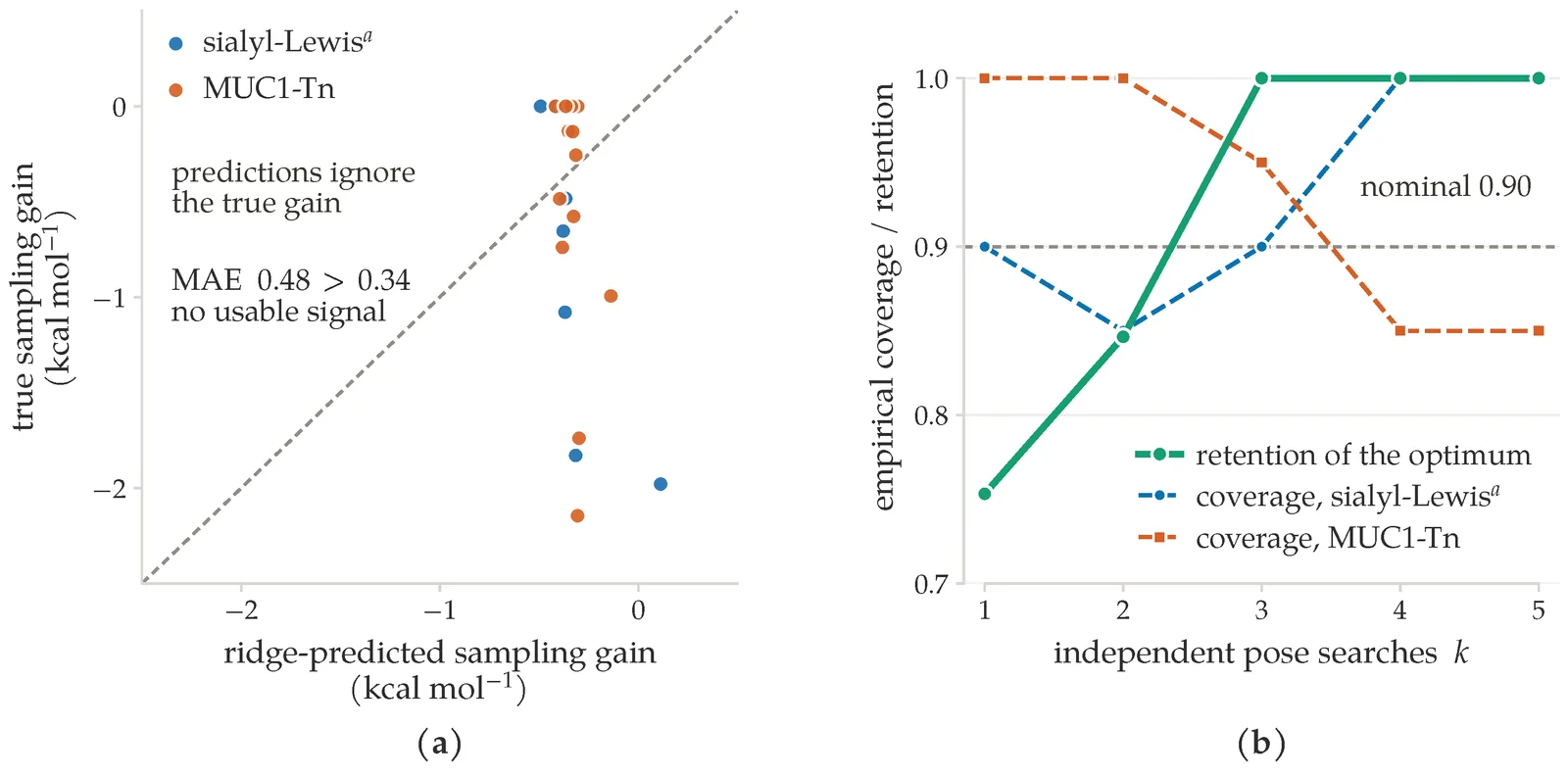
The machine learning verdict, prediction against certification on the same task. (**a**) plots the ridge-regression prediction of the residual sampling gain against its true value; the dashed line is the perfect predictor, and the points scatter far from it, the regressor reaching an MAE of 0.48 kcal mol^−1^ against a zero-gain baseline of 0.34 kcal mol^−1^, so point prediction carries no usable signal; (**b**) plots the empirical coverage of the certified set and the retention of the true optimum against the number of independent pose searches *k*; the coverage stays close to the nominal ninety-percent level, within the fluctuation expected from the small calibration group, while the retention of the true optimum reaches unity for k≥3. The distribution-free certificate thus remains informative in the same regime where point prediction of the residual fails.

The preceding subsections can now be read as a single controlled comparison between the two ways machine learning might be brought to bear on this problem, and the comparison is decisive. Figure 10 places the two side by side on the same task and the same data. The first way is to learn the quantity of interest and act on the prediction. When that route is taken here, it fails outright, as Figure 10a shows: the leave-one-out ridge regressor trained on molecular and geometric descriptors to predict the residual sampling gain confines its predictions to a narrow band that ignores the true gain, reaching an MAE of 0.48 kcal mol^−1^ that is worse than the trivial baseline of predicting zero, at 0.34 kcal mol^−1^. Gradient boosting and the addition of geometry features do not recover a usable signal, as recorded in Table S4. The second way is to abandon prediction and calibrate a distribution-free guarantee around the cheap physics, and when that route is taken it succeeds, as Figure 10b shows: the empirical coverage tracks the nominal ninety-percent level and the retention of the true optimum reaches unity for k≥3, with no accurate point predictor anywhere in the loop. The verdict of the study is contained in the contrast between these two outcomes. In the low-data regime that governs pre-synthetic screening, the dependable machine learning contribution is not a prediction but a certificate, and the framework is built to deliver the second rather than the first. This inversion, from a model that estimates a value to a protocol that guarantees a decision, is what we propose machine learning should contribute wherever the data are too scarce to train a trustworthy predictor; yet, a cheap, physically grounded surrogate is available to be certified. In that setting the honest machine learning result is not a better guess but a bounded one, and it is the bound, not the guess, that a laboratory can safely act on.

## 4. Discussion

The results of the previous section are quantitative, but their significance lies less in any single number than in the way the numbers were obtained and in what they are now licensed to support. Three findings ask to be interpreted together. A competition-corrected physics score reorders the library and isolates a small set of credible monomers; a distribution-free layer converts that ordering into a candidate set that provably retains the optimum; and a learned point predictor, offered the same information, fails to beat a trivial baseline. The discussion that follows reads these findings as one argument. It first sets out why certification, rather than prediction, is the defensible use of machine learning in this regime, then examines the statistical mechanism that makes the guarantee tight, then asks whether the chemistry the screen selects is robust enough to carry the weight that the guarantee places on it and finally situates the whole construction against the computational resources that it saves and the prior work it extends. Each subsection isolates one link in that chain so that the strength of the conclusion can be judged link by link rather than taken as a whole.

### 4.1. Certification Rather than Prediction

The results invite a reading of monomer selection as a certification problem rather than a prediction problem, and the distinction is more than rhetorical. The regression experiments of Section 3.7 show that the residual error of the cheap physics estimator is not recovered by any model we tried, since none beat a zero-gain baseline. We are careful about how far this negative result reaches. It is established on a small sample and a finite set of descriptors, so it demonstrates that point prediction is unreliable in the regime that matters here, namely tens of expensive evaluations rather than thousands, and not that the residual is intrinsically unlearnable. That qualification granted, the practical consequence stands. A pipeline that staked its decisions on a learned property value would inherit this failure in silence, presenting a confident ranking with no signal that it is unreliable. The conformal layer makes a different and more modest claim. It does not predict the residual, it bounds it, and it turns that bound into a candidate set with a finite-sample guarantee. Where the physics supplies a partially trusted energy and the statistics supply a calibrated envelope around it, the two together support a decision that neither could justify alone.

It is equally important to be clear about what the guarantee does not assert. Theorem 1 bounds the sampling error of the competition-corrected score; it does not certify that the score is chemically correct. The certificate says that, with controlled probability, the best-scoring monomer under our own metric is kept in the reported set, and its worth for the laboratory is therefore only as good as the metric it protects. This is why the cross-method agreement of Section 3.6 and the weight-sensitivity analysis of Section 3.5 are not decoration but load-bearing evidence, since they test the metric itself rather than the statistics wrapped around it.

This reading places the work squarely within the movement toward trustworthy and uncertainty-aware machine learning, but it inverts the usual order of operations. The dominant instinct in data-driven chemistry is to learn the target property and then, if resources allow, to attach an uncertainty estimate to the learned value. In the regime that governs pre-synthetic screening, where each label costs a structure optimization and the whole campaign affords tens rather than thousands of them, that order fails at the first step, because there are simply not enough labels to learn the residual well. Conformal prediction is attractive here precisely because its validity does not depend on the accuracy of any model or on the size of the training set; it needs only exchangeability, and it returns a set whose coverage is guaranteed at finite sample size. We are therefore not using the statistics to rescue a weak predictor. We are declining to build the predictor at all, and spending the same scarce evaluations on a calibrated envelope that a decision can be safely rested on. The negative regression result is not an embarrassment to be explained but the empirical fact that makes this choice the correct one.

### 4.2. Why the Guarantee Is Tight

The one-sided exactness of Lemma 1 is what makes the certificate tight rather than merely valid, and its content is elementary. Because the competition terms are evaluated once and held fixed, they contribute a constant to the estimator, so the sampling variation of $S_{\mathrm{comp}}$ is identical to that of $\Delta E_{T-M}$. Simple as this observation is, its consequence is not trivial, because it collapses the usual two-sided conformal interval to a one-sided certificate on the lower endpoint alone. The reduction pays off in the budget analysis of Section 3.8, since certifying only the side that can move is what lets the procedure stop early and still retain the optimum. Proposition 2 sharpens the same point by tracing retention to a single per-candidate event, so the radius is not inflated by the size of the library. The boundary of the argument should be stated plainly in turn. The upper side is exact only insofar as the competition terms are genuinely converged, a condition we verified numerically for this construction, and the property is not a license to ignore convergence in a different one.

It is worth naming the exact assumption on which the whole edifice rests, because it is a single one and it is testable. Split-conformal validity requires only that the calibration residuals and the residual of the monomer being tested be exchangeable, a condition far weaker than independence and far weaker than any distributional form. Here, the residuals are the sampling errors of the best-of-*k* estimator, and exchangeability within a target results in the unremarkable claim that the difficulty of sampling one monomer’s best pose is drawn from the same pool as the others. The group-conditional construction we use makes this assumption local to each epitope, so a target whose poses are systematically harder to sample cannot silently inflate the confidence of an easier one. The cross-target experiment of Section 3.7 then probes the stronger claim that residuals are exchangeable across epitopes as well, and that a radius calibrated on one target covers the other is direct, if limited, evidence that the sampling error has a shape of its own that is largely independent of the chemistry it is measured on. That the guarantee reduces to one checkable premise, rather than to a chain of modeling assumptions, is the deeper reason to prefer it.

### 4.3. Robustness of the Selected Chemistry

A guarantee about sampling error is only as valuable as the score it protects, so the argument now turns from the statistics to the chemistry and asks a blunt question: would the same two monomers be selected if the modeling choices had been made differently? Two independent analyses, one varying the free parameters of the score and one varying the electronic structure method entirely, answer that they would.

The weight sensitivity of Section 3.5 shows that the identification of EDOT and 3-APBA is stable across almost the entire (α,β) square, with EDOT never leaving the top two positions and 3-APBA losing the lead only where the competition penalties are effectively removed, as Figure 6 shows. The cross-method check of Section 3.6 shows that the ordering and, crucially, the sign of the EDOT selectivity survive a change of tight-binding Hamiltonian, as Figure 7 shows. A first-principles check places the boron–diol recognition of the Tn sugar on a firmer footing: at the gas-phase PBE-D3(BJ) level the interaction energy is −13.2 kcal mol^−1^ in the larger def2-TZVP basis, some 3.8 kcal mol^−1^ less bound than in def2-SVP once basis-set superposition error is removed, but still strongly favorable. That absolute energies are this sensitive to the basis is exactly why selectivity is judged throughout from consistently computed energy differences rather than absolute values, and why the reliability of a selectivity is governed by the pose sampling of Section 3.4, not by the level of theory. Taken together, the central chemical claims rest on features of the interaction that are shared by independent levels of theory and are insensitive to the one free parameter of the score.

These claims also agree with the experimental record, which is a necessary sanity check on any in silico screen. EDOT is a workhorse of electropolymerized sensor films, and its emergence here as the affinity optimum for the CA 19-9 epitope rationalizes that empirical popularity on structural grounds [26,27]. Aminophenylboronic acids are established receptors for the cis-diols of sialylated glycans, which matches the identification of 3-APBA as the MUC1-Tn optimum and the certified selectivity of its close analog 4-APBA for the Tn sugar, consistent with the wider understanding of boronic acid recognition of saccharides [28]. That the same screen also flags the sialyl-Lewis^x^ regioisomer discrimination as under-determined, rather than passing off an under-sampled gap as a decisive hit, is exactly the honest prioritization a pre-synthetic screen should deliver. The competition correction earns its place in this outcome, since it removes acrylamide, a strong but promiscuous self-associator, from the top of the list and so prevents a monomer that would imprint poorly from consuming experimental effort.

### 4.4. Practical Implications and Relation to Prior Work

If the statistics are tight and the chemistry is robust, the final question is what the combination buys a working laboratory that the established approaches do not, and where it sits in the arc of computational design for imprinted polymers. Two things distinguish it: a calibration that is spent on the error rather than on the target, and a deliverable that is a guaranteed set rather than a ranked list.

The portability recorded in Empirical Observation 1 is where a purely statistical device meets the chemistry. A quantile calibrated on one glycoepitope still retains the optimum on the other, at empirical coverage close to the nominal level, which means the calibration is spending its information on the shape of the sampling error rather than on the identity of the target. For a laboratory that screens a family of related analytes, this is the difference between recalibrating for every target and reusing one calibration across a panel of targets. Set against prior computational MIP design, which has advanced from single-energy monomer ranking to broad simulation-aided and data-driven workflows [20,21,22], the novelty here is not another score but a change in what the screen returns. Instead of a ranked list taken on faith, it returns a set with a coverage guarantee and a compute budget that the guarantee itself decides, cutting tight-binding effort at equal retention. The combination of a competition-corrected physics score with distribution-free certification, and the observation that the correction makes one side of the estimator error exact, is to our knowledge new to this domain.

Scaling to a larger candidate library is governed by where the cost actually lies. The pose search is performed once per template and monomer pair and never compares monomers to one another, so the tight-binding effort grows linearly with the number of candidates; a fivefold larger library multiplies that effort roughly fivefold and nothing worse, and the best-of-*k* stopping rule adds only a constant per-candidate overhead rather than a term that grows with the library. The certification layer is cheaper still, since its cost is set by the size of the calibration group and not by the number of candidates screened, so enlarging the library leaves the conformal computation essentially untouched. What does not scale for free is the statistics rather than the arithmetic: as the library widens the calibration group must stay representative of the chemistry being ranked, and it is the breadth of that group, not the compute, which bounds how far a single certificate can be stretched. Because the per-candidate cost is a fraction of a CPU-hour of tight binding and the certificate is almost free, a library of several hundred monomers remains within reach of a modest local cluster, and the low arithmetic cost is what would make a high-throughput, in-house deployment practical rather than requiring specialized infrastructure.

The practical horizon this opens is broader than the two receptors used to demonstrate it. Because the conformal layer treats the energy as an oracle to be bounded rather than a model to be trusted, every component beneath it can be upgraded without touching the guarantee: a denser decoy panel, a hybrid-functional energy, or a free-energy estimate from explicit-solvent sampling would each sharpen the physics while the coverage statement stood unchanged. The same indifference to the underlying generator is what makes an eventual union with generative monomer design natural rather than forced, since a proposal engine and a certifier exchange only candidates and residuals. Seen this way, the contribution is less a finished screen for two glycoepitopes than a reusable discipline for spending scarce, expensive computation under an explicit and auditable confidence budget, and it is in that reusability, as much as in the specific monomers it flags, that we locate the value of the work.

## 5. Limitations

The framework has real limitations, and we set them out without softening so that its claims are not read more widely than intended. They fall into two kinds. The first concerns the physics: the energies are a fast, approximate surrogate for a true binding free energy, and the guarantee the method provides is a statement about sampling, not about the fidelity of that surrogate, so a systematic error in the energy model would be certified as confidently as a correct one. The second concerns the reach of the claim, both the statistical scope of the conformal guarantee under small and structured calibration groups and the physical scope of a recognition model that stops at the monomer-and-template stage and is silent on the polymerization, morphology, and rebinding steps a working sensor must still negotiate. The subsections below take these in turn, separating throughout what the certificate guarantees from what it leaves to experiment. Stating these boundaries precisely is itself part of the method’s claim to trustworthiness, since a guarantee is credible only when the conditions under which it can fail are named as plainly as the conditions under which it holds.

### 5.1. The Physical Model

The energies are semiempirical tight-binding values, not density-functional energies and not free energies from explicit-solvent molecular dynamics, so absolute affinities should be read as a consistent ordering and not as thermodynamic quantities. Entropy, explicit solvent, and conformational averaging beyond the pose search are absent, and the implicit ALPB treatment is a coarse description of the strongly hydrated, and in the case of the sialic acid formally charged, glycans studied here, which were treated at a single neutral protonation state for consistency across methods. We checked that this does not overturn the robust recognition by re-screening the leading monomers against the CA 19-9 epitope with its sialic-acid carboxylate deprotonated to the physiological −1 charge, reusing the charge-independent competition terms and recomputing only the template-monomer interaction. The certified selection is unchanged: EDOT and 3-APBA remain the two highest-ranked monomers, with competition-corrected scores of −12.7 and −12.1 kcal mol^−1^ on the anionic epitope, if anything more favorable than their neutral values of −8.3 and −7.8, so deprotonation strengthens rather than overturns the recognition. EDOT still favors the sialylated epitope over the non-sialylated Lewis^a^ core by 6.8 kcal mol^−1^, so the sialylation recognition that defines the determinant survives the charge, while the harder sialyl-Lewis^x^ regioisomer gap remains in the under-determined range found for the neutral state, and the full re-screen of the leading monomers is reported in Table S5. The Tn-glycosylation recognition is intrinsically unaffected, since the MUC1-Tn glycopeptide carries no ionizable carboxylate at physiological pH. The competition correction represents the competing equilibria through pairwise surrogates rather than a full pre-polymerization equilibrium, and its equal weighting of the two penalties is a modeling decision. The sensitivity analysis of Section 3.5 shows the conclusions are robust to that decision, but it does not make the weighting first-principles.

### 5.2. The Scope of the Guarantee

Beyond the fidelity of the energy just discussed, the guarantee carries statistical limits of its own that set how far a single certificate may be trusted. The calibration groups are small, on the order of twenty residuals, which makes the empirical quantile coarse and the nominal ninety-percent level granular in practice. The cross-target transfer of Empirical Observation 1 rests on an exchangeability assumption that we verify empirically, but only in the two directions afforded by two epitopes, so it should be regarded as encouraging evidence rather than a general law. As a first control on the group size, pooling the residuals across both epitopes and all monomers doubles the calibration group to n=40 and yields a radius of 1.74 kcal mol^−1^ at ninety-percent confidence, essentially the same threshold as the per-target groups and reproducing the certified sets, which indicates the guarantee is not an artifact of the smaller group; a fuller test across many targets and decoys is the natural next refinement. A sharper limit is exchangeability across chemical families: the residuals of the classical acrylic monomers are significantly larger than those of the electropolymerizable set, as Section 3.7 reports, so a pooled calibration is mildly anticonservative when a radius trained on one family is applied to the other, with leave-one-class-out coverage falling to 0.625 in the worst case. With only four monomers in the minority family a family-conditional quantile is not finitely estimable at ninety-percent confidence, so pooling is the pragmatic choice here; a library balanced across families would let the group-conditional construction remove this residual anticonservatism.

### 5.3. Scope of the Model and the Negative Result

The framework describes molecular recognition at the monomer-and-template stage, which is where selectivity is decided, and it does not attempt to model the steps that follow. Cross-linking, polymer morphology, template removal, and rebinding kinetics lie outside its scope, so its output is a certified candidate set and a selectivity assessment, not a predicted imprinting factor or detection limit, and it should be read as the prioritization layer it is designed to be. The decoy panel is deliberately compact and centered on the hardest isomer for each target, which is enough to expose the selectivity problem; each selectivity value now carries a multi-seed resampling interval rather than standing as a bare number, but it remains a differential energy for a single decoy pair rather than a full specificity profile over many interferents. The negative regression result of Section 3.7, finally, is bounded by the descriptors and the data examined; it motivates certification over prediction in this low-data regime and should not be generalized beyond it.

### 5.4. From Certified Shortlist to Experimental Validation

A certified shortlist is a pre-synthetic hypothesis, not a statement of device performance, and the natural end of the workflow is therefore the laboratory rather than the computer. The guarantee of Section 2.6 concerns the sampling error of a competition-corrected tight-binding score; it says nothing about the adsorption capacity, the imprinting factor, or the real-sample selectivity that only synthesis and rebinding assays can establish. Two sources of complexity sit entirely outside the model and must be settled experimentally. The first is the measurement environment. The screen reasons over isolated glycoepitope fragments, whereas a deployed sensor meets whole glycoproteins in a serum matrix whose abundant background proteins compete for the surface and can foul it, so a recognition event demonstrated in silico is a starting point whose selectivity against real interferents has to be confirmed in biological fluid and, where necessary, protected by antifouling design [41]. Because the framework ingests no experimental binding data, it neither depends on nor is undermined by the scatter of such measurements; it is built for exactly the data-scarce regime in which reliable experimental affinities are not yet available, and it returns a prioritized set to be measured rather than a measurement to be trusted.

The second source of complexity is the polymer itself. The framework fixes the single most consequential and least reversible decision, the identity of the functional monomer, but it is silent on the monomer-to-template stoichiometry, the cross-linking density, the porogen, and the polymerization and template-removal steps that together set the number and the uniformity of the cavities finally formed. Modest changes in these conditions can broaden the distribution of binding-site affinities and shift the measured performance, an effect that is quantified experimentally through the heterogeneous affinity distribution of the cured material [42], so the certified monomer is best read as the entry point to a downstream experimental optimization rather than its conclusion. Seen in this light, the value of the screen is that it contracts an expensive combinatorial search to a small and guaranteed set before any monomer is polymerized, turning an open-ended trial of candidate chemistries into a focused program in which the computational and experimental descriptions of the same system can be compared and reconciled directly [43]. The framework is thus a prioritization layer designed to be validated, and the experiments it motivates, rather than any single monomer it certifies, are where its predictions are ultimately tested.

## 6. Conclusions

We set out to make functional-monomer selection for molecularly imprinted polymers at once competition-aware and trustworthy, and we achieved this by recasting the choice as a certification problem rather than a prediction problem. The outcome is, to our knowledge, the first monomer screen that issues a finite-sample, distribution-free guarantee on the retention of the optimal monomer. Coupling GFN2-xTB pose energies, a competition-corrected affinity score, and a best-of-*k* estimator to a conformal bound yields a candidate set that provably contains the optimum with controlled probability, and by exploiting the one-sided exactness of the corrected estimator the procedure attains this without any correction for the size of the library and with a threshold that, in our data, carries across related targets. Lemma 1 and Proposition 2 make the first two of these claims exact, while the transfer across related targets is the empirical content of Empirical Observation 1 rather than a theorem.

The framework proves its worth on two clinically important pancreatic cancer glycoepitopes. Applied to sialyl-Lewis^a^, the CA 19-9 determinant, and to MUC1-Tn, it contracts a twenty-monomer library to two and five certified candidates at ninety-percent confidence, cuts the tight-binding compute by thirty-five to sixty-seven percent, and identifies EDOT and 3-APBA as certified affinity optima. The multi-seed certificate then separates the selectivity claims that are sampling-robust, EDOT reading the sialylation of sialyl-Lewis^a^ and 4-APBA reading the Tn-glycosylation of MUC1-Tn, from the harder sialyl-Lewis^x^ regioisomer discrimination, which it flags as under-determined at the available pose budget rather than reporting it as a decisive hit. The affinity selection is stable across almost the entire space of competition weights, and the interaction energies are reproduced across two tight-binding parametrizations and a density-functional single point; the reliability of a selectivity, by contrast, is a question the certificate answers directly, and on the hardest isomer task it returns an honest negative, which is itself the kind of verdict the method exists to deliver.

The significance of this outcome extends beyond the two receptors. Functional-monomer choice has long been the least principled step in imprinted-polymer design, settled by trial libraries and single-energy rankings that offer no measure of their own reliability, and it is that silence regarding reliability rather than any lack of candidate chemistries that has held back the rational design of synthetic recognition. By returning a set with a stated coverage and a compute budget the coverage itself fixes, the framework replaces an unquantified judgment with an auditable one. For targets as demanding as the sialylated glycans of CA 19-9 and MUC1-Tn, where the discriminating difference is a single regiochemical isomer and a misplaced trust would cost months of synthesis, a quantified assurance that the score-optimal monomer has not been discarded is a practical safeguard rather than a statistical nicety and is the difference between a screen worth acting on and one that is not.

The methodological lesson is carried by a single sharp contrast. No point prediction model we tried beat a trivial baseline, while certification succeeded, and the two facts together say something specific about how machine learning should be used with cheap physics in a low-data regime. The reliable contribution there is not a learned value but a guarantee around a value one already partly trusts. This reframing is general. It applies wherever an inexpensive, partially trusted computation must drive an expensive decision, and it turns the usual question—how accurate is the model—into a more answerable one: how confident is the decision.

These findings place functional-monomer selection, for the first time, on a footing where the confidence of the choice is computed rather than assumed. The wider extensions this opens, to larger libraries, to higher-fidelity energy models, and to a union with generative design in which candidates are proposed and certified together, all follow from the single principle that Section 4.4 develops in full: an inexpensive and only partly trusted physics can responsibly drive an expensive experimental decision whenever a distribution-free certificate stands between the computation and the commitment. That principle, more than any particular monomer the screen certifies for CA 19-9 and MUC1-Tn, is the lasting contribution of this study, and it is what recommends physics-constrained conformal screening as a standard for trustworthy, resource-aware molecular design well beyond the recognition elements examined here.

## Figures and Tables

**Table 1:** The twenty-monomer library, giving the numbers of hydrogen bond donors (HBD) and hydrogen bond acceptors (HBA).

Monomer	Abbreviation	Class	Functional Groups	HBD	HBA
2-aminophenol	o-AP	functional	amine, phenol	2	2
2-aminophenylboronic acid	2-APBA	functional	boronic acid, amine	3	3
3,4-ethylenedioxythiophene	EDOT	functional	thiophene, dialkyl ether	0	3
3-aminophenylboronic acid	3-APBA	functional	boronic acid, amine	3	3
4-aminobenzoic acid	PABA	functional	amine, carboxylic acid	2	2
4-aminophenylboronic acid	4-APBA	functional	boronic acid, amine	3	3
4-aminothiophenol	4-ATP	functional	thiol, amine	2	2
4-vinylphenylboronic acid	4-VPBA	functional	boronic acid, vinyl	2	2
aniline	aniline	functional	aromatic amine	1	1
dopamine	dopamine	functional	catechol, amine	3	3
gallic acid	gallic acid	functional	pyrogallol, carboxylic acid	4	4
norepinephrine	norepinephrine	functional	catechol, amine, alcohol	4	4
o-phenylenediamine	o-PD	functional	aromatic diamine	2	2
phenol	phenol	functional	phenol	1	1
pyrrole	pyrrole	functional	N–H donor, π system	1	0
scopoletin	scopoletin	functional	coumarin, phenol, ether	1	4
2-hydroxyethyl methacrylate	HEMA	reference	hydroxyl, ester	1	3
4-vinylpyridine	4-VP	reference	pyridine, vinyl	0	1
acrylamide	acrylamide	reference	primary amide	1	1
methacrylic acid	MAA	reference	carboxylic acid, vinyl	1	1

**Table 2:** The five highest-ranked monomers per epitope by $S_{\mathrm{comp}}$. Energies are in kcal mol^−1^, and *k* is the number of independent pose searches pooled for that complex.

Epitope	Monomer	$\Delta E_{T-M}$	$\Delta E_{M-M}$	$\Delta E_{M-S}$	$S_{\mathrm{comp}}$	Rank ΔE	Rank $S_{\mathrm{comp}}$	*k*
sialyl-Lewis^a^	EDOT	−11.80	−5.20	−1.81	−8.30	1	1	4
sialyl-Lewis^a^	3-APBA	−10.24	−2.89	−2.08	−7.76	3	2	5
sialyl-Lewis^a^	scopoletin	−9.52	−3.47	−2.96	−6.30	4	3	5
sialyl-Lewis^a^	acrylamide	−11.23	−8.87	−2.82	−5.39	2	4	5
sialyl-Lewis^a^	o-AP	−8.45	−3.75	−3.08	−5.03	6	5	5
MUC1-Tn	3-APBA	−8.02	−2.89	−2.08	−5.54	5	1	4
MUC1-Tn	4-APBA	−8.78	−5.45	−2.97	−4.57	4	2	12
MUC1-Tn	o-PD	−6.63	−1.93	−2.60	−4.36	8	3	5
MUC1-Tn	acrylamide	−10.14	−8.87	−2.82	−4.30	1	4	6
MUC1-Tn	scopoletin	−7.41	−3.47	−2.96	−4.19	6	5	5

Ranks are taken within each epitope over the full twenty-monomer library.

**Table 3:** The pose-budget analysis. The mean and the dispersion (standard deviation, SD) of the ensemble interaction energy, across seeds and within pairs, are given in kcal mol^−1^.

Budget	Poses Generated	Poses Optimized	Time per Search (s)	Mean $\Delta E_{\mathrm{ens}}$	SD Across Seeds	SD Within Pair
low (B1)	120	6	101	−7.52	2.05	1.82
medium (B2)	1000	10	212	−9.04	1.58	1.61
high (B3)	3000	16	369	−9.80	1.40	0.97

## Data Availability

The original contributions presented in this study are included in the article. Further inquiries can be directed to the corresponding author.

## Supplementary Materials

The following supporting information can be downloaded at: https://www.mdpi.com/article/10.3390/polym18212699/s1, Figure S1: the stoichiometry analysis of the leading monomers. Table S1: the full competition-corrected scores for all template and monomer pairs. Table S2: the structure inventory with stereochemistry verification. Table S3: the full differential-binding matrix with multi-seed intervals. Table S4: the machine learning regression baselines for the residual sampling gain. Figure S2: the best-of-k convergence of the sialyl-Lewis regioisomer pair under equalized sampling. Table S5: the competition-corrected re-screen of the leading monomers with the sialic-acid carboxylate of the CA 19-9 epitope deprotonated to its physiological anionic state.

## Abbreviations

The following abbreviations are used in this manuscript:

AI	artificial intelligence
MIP	molecularly imprinted polymer
CA 19-9	carbohydrate antigen 19-9, the sialyl-Lewis^a^ epitope
MUC1	mucin 1
MUC1-Tn	MUC1 bearing the truncated Tn O-glycan
GFN2-xTB/GFN1-xTB	geometry, frequency, noncovalent tight-binding method, second/first generation
ALPB	analytical linearized Poisson–Boltzmann solvation model
PBE	Perdew-Burke-Ernzerhof exchange-correlation functional
def2-SVP/def2-TZVP	split-valence/triple-zeta-valence polarized def2 basis set
D3(BJ)	Grimme D3 dispersion with Becke-Johnson damping
ETKDG	experimental-torsion knowledge distance geometry
MMFF	Merck molecular force field
RCSB	Research Collaboratory for Structural Bioinformatics
$S_{\mathrm{comp}}$	competition-corrected affinity score
ΔE	site-directed interaction energy
ΔΔE	differential binding energy, target minus decoy
${\hat{S}}_{k}$	best-of-*k* pose estimator
EDOT	3,4-ethylenedioxythiophene
3-APBA/4-APBA	3-/4-aminophenylboronic acid
MAE	mean absolute error
SD	standard deviation
CPU	central processing unit
HBD/HBA	hydrogen bond donor/acceptor
