# Supplementary material for: Physics-Constrained Conformal Screening: A Certified Machine Learning Framework for the Rational Design of Dual Glycoepitope-Imprinted Polymers Recognizing CA 19-9 and MUC1-Tn
Source: Polymers (Basel). 2026 Oct 31;18(21):2699. doi: 10.3390/polym18212699 (PMC13765833; doi:10.3390/polym18212699)
Supplement: Supplementary file 1 [file polymers-18-02699-s001.zip › polymers-4636552-supplementary.pdf]

Supplementary Materials

Physics-Constrained Conformal Screening: A Certified Machine-Learning Framework for the Rational Design of Dual Glycoepitope-Imprinted Polymers Recognizing CA 19-9 and MUC1-Tn

Yetkin Senemoğlu and Aytaç Altan

This document contains the supporting data referenced in the main text. All values are the direct output of the computational pipeline (GFN2-xTB / GFN1-xTB with ALPB(water) solvation, PBE-D3(BJ) single points, and split-conformal prediction); no values are rounded beyond the precision shown. The competition-corrected score used to rank the monomers is

with , where is the template–monomer interaction energy, the monomer self-association energy, and the monomer solvation-surrogate energy, exactly as in Definition 2 of the main text.

Table S1. Competition-corrected screening data for all twenty candidate monomers against both epitopes. is the template–monomer interaction energy with sialyl-Lewis (SLe) and with MUC1-Tn; is the monomer self-association energy and the monomer solvation-surrogate energy (both target-independent); and is the resulting competition-corrected score for each epitope. All energies are in kcal mol and are best-of- pooled estimates; rows are ordered by against sialyl-Lewis.

Monomer	ΔE_{T-M} (SLe^a)	ΔE_{T-M} (Tn)	ΔE_{M-M}	ΔE_{M-S}	S_{comp} (SLe^a)	S_{comp} (Tn)
EDOT	−11.80	−5.78	−5.20	−1.81	−8.30	−2.27
3-APBA	−10.24	−8.02	−2.89	−2.08	−7.76	−5.54
scopoletin	−9.52	−7.41	−3.47	−2.96	−6.30	−4.19
acrylamide	−11.23	−10.14	−8.87	−2.82	−5.39	−4.30
o-AP	−8.45	−6.30	−3.75	−3.08	−5.03	−2.89
aniline	−6.67	−5.80	−2.51	−2.60	−4.12	−3.25
phenol	−5.51	−5.49	−2.07	−1.26	−3.84	−3.82
o-PD	−5.96	−6.63	−1.93	−2.60	−3.69	−4.36
gallic acid	−9.20	−6.20	−7.52	−3.75	−3.56	−0.56
2-APBA	−7.57	−6.47	−6.31	−1.89	−3.47	−2.37
4-APBA	−7.59	−8.78	−5.45	−2.97	−3.38	−4.57
4-VPBA	−5.98	−5.99	−4.46	−2.08	−2.71	−2.71
4-ATP	−5.01	−5.77	−3.23	−1.58	−2.61	−3.36
HEMA	−6.26	−4.88	−5.16	−2.42	−2.47	−1.09
4-VP	−4.96	−6.65	−4.49	−1.12	−2.15	−3.84
pyrrole	−5.40	−4.93	−2.91	−3.68	−2.10	−1.64
norepinephrine	−4.25	−5.14	−2.62	−1.93	−1.98	−2.87
dopamine	−4.46	−5.41	−3.18	−2.09	−1.82	−2.78
MAA	−7.15	−8.93	−9.01	−3.74	−0.77	−2.56
PABA	−7.24	−9.25	−8.92	−4.19	−0.69	−2.70

Table S2. Structure inventory for the two template epitopes and the four decoy glycans, with the molecular formula, the number of defined stereocenters, and the InChIKey used to verify anomeric configuration and glycosidic linkage against the RCSB Chemical Component Dictionary. The twenty candidate monomers and their polymerizable classes are listed in Table 1 of the main text.

Structure	Role	Formula	Stereo-centers	InChIKey
sialyl-Lewis^a (CA 19-9 epitope)	Template	$C_{31}H_{52}N_2O_{23}$	21	XBSNXOHQOTUENA-UQTBNESHSA-N
MUC1-Tn core (GalNAc-a-O-Thr)	Template	$C_{14}H_{25}N_3O_8$	7	UCYZYYFONADRFX-LKAFSPJPSA-N
sialyl-Lewis^x (positional isomer decoy)	Decoy	$C_{31}H_{52}N_2O_{23}$	21	NIGUVXFURDGQKZ-UQTBNESHSA-N
Lewis^a (desialylated decoy)	Decoy	$C_{20}H_{35}NO_{15}$	15	CFDVGUXRLQWLJX-LPHOMBEVSA-N
Lewis^x (desialylated decoy)	Decoy	$C_{20}H_{35}NO_{15}$	15	HBBOZFUQJDYASD-LPHOMBEVSA-N
N-acetyl-L-threoninamide (aglycone decoy)	Decoy	$C_6H_{12}N_2O_3$	2	APGCJAUAUXZOPI-WUJLRWPWSA-N

Table S3. Full differential-binding matrix. For each (target, monomer, decoy) claim, and are the best-of- interaction energies and (kcal mol); a negative favors the target. For the six claims carried to a multi-seed certificate, the 95% distribution-free resampling interval is given; a dash marks claims not carried to resampling. A claim is sampling-robust only when its resampling interval lies entirely below zero.

Target	Monomer	Decoy	ΔE_{target}	ΔE_{decoy}	$\Delta\Delta E$	95% CI
sialyl-Lewis^a	3-APBA	Lewis^a	−10.24	−4.32	−5.93	—
sialyl-Lewis^a	EDOT	Lewis^a	−11.80	−5.41	−6.40	[−7.90, −1.33]
sialyl-Lewis^a	acrylamide	Lewis^a	−11.23	−7.00	−4.23	—

Target	Monomer	Decoy	ΔE_{target}	ΔE_{decoy}	$\Delta\Delta E$	95% CI
sialyl-Lewis^{a}	o-AP	Lewis^{a}	−8.45	−4.36	−4.09	—
sialyl-Lewis^{a}	scopoletin	Lewis^{a}	−9.52	−5.04	−4.48	—
sialyl-Lewis^{a}	3-APBA	Lewis^{x}	−10.24	−3.45	−6.79	—
sialyl-Lewis^{a}	EDOT	Lewis^{x}	−11.80	−8.70	−3.10	[−7.79, 1.58]
sialyl-Lewis^{a}	acrylamide	Lewis^{x}	−11.23	−8.75	−2.48	—
sialyl-Lewis^{a}	o-AP	Lewis^{x}	−8.45	−5.04	−3.42	—
sialyl-Lewis^{a}	scopoletin	Lewis^{x}	−9.52	−8.56	−0.96	—
sialyl-Lewis^{a}	3-APBA	sialyl-Lewis^{x}	−10.24	−9.92	−0.33	[−3.77, 5.05]
sialyl-Lewis^{a}	EDOT	sialyl-Lewis^{x}	−11.80	−8.38	−3.43	[−4.35, 1.64]
sialyl-Lewis^{a}	acrylamide	sialyl-Lewis^{x}	−11.23	−11.20	−0.04	—
sialyl-Lewis^{a}	o-AP	sialyl-Lewis^{x}	−8.45	−11.95	3.50	—
sialyl-Lewis^{a}	scopoletin	sialyl-Lewis^{x}	−9.52	−9.93	0.41	—
MUC1-Tn	3-APBA	N-Ac-Thr	−8.02	−6.47	−1.55	[−4.39, 1.34]
MUC1-Tn	4-APBA	N-Ac-Thr	−8.78	−6.72	−2.06	[−3.03, −0.56]
MUC1-Tn	acrylamide	N-Ac-Thr	−10.14	−9.80	−0.34	—
MUC1-Tn	o-PD	N-Ac-Thr	−6.63	−6.74	0.11	—
MUC1-Tn	scopoletin	N-Ac-Thr	−7.41	−5.11	−2.30	—

Table S4. Machine-learning regression baselines for the residual sampling gain that remains after independent pose searches. Mean absolute error (MAE, kcal mol) of ridge regression and gradient-boosted trees (GBM), trained on molecular descriptors alone and with added geometric descriptors, against the trivial baseline that predicts zero residual gain. is the number of (template, monomer) pairs still active at that , which falls from forty at to thirty-nine at as a pair converges; the conformal calibration of the main text instead uses the full forty-pair group, so the two counts refer to different objects. No learned model beats the baseline, which is why the framework certifies with a distribution-free conformal bound rather than a point prediction, as Section 3.7 and Figure 8c of the main text develop.

Searches k	n	Baseline (no gain)	Ridge (desc.)	GBM (desc.)	Ridge (+ geom.)	GBM (+ geom.)
2	40	1.014	1.287	1.293	1.197	1.262
3	39	0.339	0.459	0.496	0.481	0.450

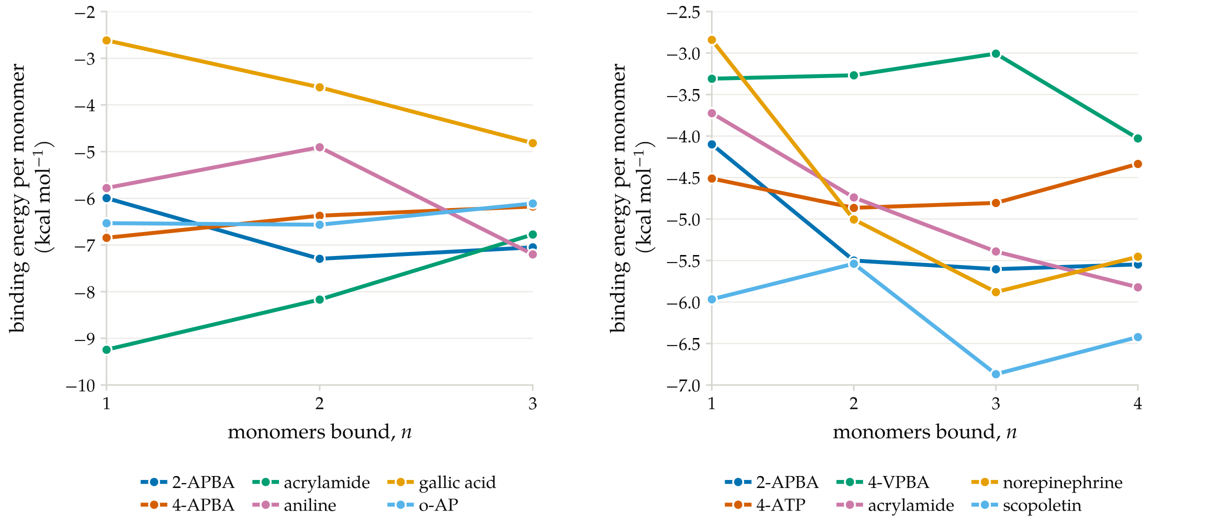

(a) (b)

Figure S1. Per-monomer binding energy () as a function of the number of monomers bound to (a) sialyl-Lewis and (b) MUC1-Tn, from GFN2-xTB optimizations of the 1:1 through 1:3 (SLe) or 1:4 (MUC1-Tn) complexes. For the leading recognition monomers the per-monomer energy stays close to its 1:1 value, confirming that the reported interactions correspond to defined one-to-one site occupation rather than to incidental additional stacking.

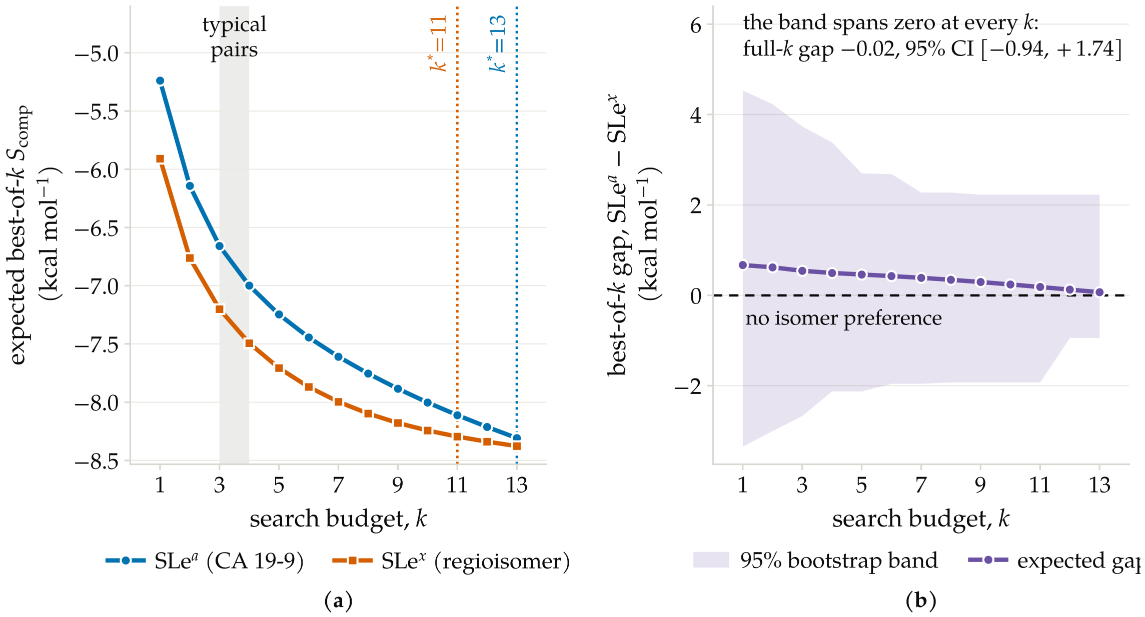

Figure S2. Best-of- convergence for the sialyl-Lewis regioisomer pair, from the extended multi-seed re-screen of EDOT against the CA 19-9 determinant sialyl-Lewis (SLe, 14 searches) and its regioisomer sialyl-Lewis (SLe, 13 searches). (a) Exact expected best-of- competition-corrected score against the search budget for each isomer; the dotted lines mark the budget at which each curve reaches within kcal mol of its plateau, and the shaded strip marks the – at which ordinary monomer–template pairs converge. (b) The best-of- affinity gap with its 95% bootstrap band; the band spans zero at every budget and the full- gap is kcal mol with a 95% interval of , so the apparent isomer preference is an artifact of finite pose sampling rather than a resolved discrimination. This is why the framework flags the regioisomer discrimination as under-determined rather than reporting it as a decisive selectivity.

Table S5. Deprotonated-state re-screen of the leading monomers against the CA 19-9 determinant. Each monomer is re-evaluated with the sialyl-Lewis sialic-acid carboxylate deprotonated to its physiological charge; the charge-independent competition terms are reused from the neutral screen, so only the template–monomer term is recomputed. and are the anionic interaction energy and competition-corrected score (kcal mol); is the shift from the neutral value. Rows are ordered by .

Monomer	$\Delta E_{T\text{-}M}$ (anion)	S_{comp} (anion)	S_{comp} (neutral)	ΔS_{comp}
EDOT	−16.19	−12.68	−8.30	−4.38
3-APBA	−14.59	−12.11	−7.76	−4.35
scopoletin	−13.81	−10.60	−6.30	−4.30
o-PD	−11.77	−9.51	−3.69	−5.82
phenol	−10.17	−8.51	−3.84	−4.67
aniline	−9.78	−7.23	−4.12	−3.11
acrylamide	−12.68	−6.84	−5.39	−1.45
o-AP	−9.61	−6.19	−5.03	−1.16

The two highest-ranked monomers are unchanged under deprotonation (EDOT and 3-APBA remain the certified top two), and the anionic scores are uniformly more favorable than the neutral ones, so the charge state strengthens rather than overturns the recognition. For the leading EDOT complex the anionic interaction energy favors the sialylated sialyl-Lewis^a epitope over the non-sialylated Lewis^a core by 6.84 kcal mol^{-1}, preserving the sialylation recognition that defines the CA 19-9 determinant.
